# A Systemic Immune State Axis Distinguishes Psoriatic Arthritis from Psoriasis

**DOI:** 10.3390/ijms27115121

**Published:** 2026-06-05

**Authors:** Yoon Kyeong Lee, Hyun-A. Seong

**Affiliations:** 1 Department of Biochemistry, College of Natural Sciences, Chungbuk National University, Cheongju 28644, Republic of Korea; ykleebio@chsu.ac.kr; 2 Department of Advanced Bio-Convergence, Chungbuk Health & Science University, Cheongju 28150, Republic of Korea

**Keywords:** psoriasis, psoriatic arthritis, systemic inflammation, immune state axis, DNA methylation, single-cell RNA sequencing, skin transcriptomics, disease axis

## Abstract

Psoriasis and psoriatic arthritis (PsA) are systemic immune-mediated diseases, but the features that distinguish cutaneous-dominant psoriasis from musculoskeletal involvement remain unclear. We analyzed four core public cross-sectional datasets spanning whole-blood methylation, PBMC single-cell RNA sequencing summarized at the subject level, skin RNA sequencing, and purified CD4^+^ T-cell methylation, and used two additional public skin cohorts for external contextual checks to define a disease inflammatory response axis (DIR) and a contrast-resolved systemic state coordinate (CRS) representing systemic immune state variation associated with PsA. In whole-blood methylation, DIR primarily separated healthy controls from psoriasis, whereas CRS separated psoriasis from PsA with minimal correlation to DIR. In untreated-only PBMC single-cell reanalysis, CRS separated PsA from PsO; the source-defined PSX subgroup (joint pain without CASPAR-classified PsA) showed heterogeneous subject-level positioning, with above-band enrichment concentrated at the cell-type level. Cell-type-resolved analyses localized CRS-related shifts to T-cell, monocyte, B-cell, and regulatory compartments and identified multicompartment pathway state remodeling along the CRS continuum. In contrast, skin RNA sequencing mainly captured lesional inflammatory burden and showed only limited additional PsA-related separation within the same tissue state. These findings support a model in which PsA is distinguished from psoriasis by an additional systemic immune state axis rather than by skin inflammatory burden alone.

## 1. Introduction

Psoriasis and psoriatic arthritis (PsA) are chronic immune-mediated disorders that are increasingly recognized as components of a broader systemic inflammatory spectrum rather than isolated organ-specific diseases [1,2,3]. Although psoriasis and PsA share substantial immunologic and inflammatory features, only a subset of patients with psoriasis develop musculoskeletal involvement [3,4]. The key unresolved question is therefore not whether psoriasis is systemic, but which additional systemic features distinguish psoriasis from PsA.

Previous studies have identified immune, metabolic, epigenetic, and tissue-level abnormalities in both psoriasis and PsA; however, many analyses have remained centered on a single tissue, a dominant molecular layer, or a limited set of pairwise contrasts, even when multimodal data were available [5,6,7,8]. These approaches are useful for identifying differentially expressed genes, methylation changes, enriched pathways, and cohort-specific classifiers, but they are less well suited to separating biological programs that are shared across psoriatic disease from those that are specifically associated with PsA. Recent integrative frameworks in psoriasis have also illustrated the value of multi-layer public data synthesis, but they have not been designed to distinguish biology shared with PsA from the systemic immune state shift associated with arthritis development [9]. In practice, skin inflammatory activity, circulating immune changes, and cell composition effects are often interpreted together, which makes it difficult to determine whether PsA represents a quantitatively stronger form of psoriasis or a qualitatively distinct systemic immune state.

Accordingly, the present study was designed less as a search for another single-layer biomarker and more as a cross-modality coordinate framework. Relative to earlier analyses emphasizing cell-type markers, machine learning classification, or purified cell methylation differences within one cohort [5,7], the specific aim here was to place blood- and skin-derived signals into a common biological structure that separates shared psoriatic inflammatory burden from the additional systemic state associated with PsA.

A framework that separates shared inflammatory disease activity from the additional biology associated with PsA would be biologically informative and clinically relevant [10,11,12]. If psoriasis and PsA share a common inflammatory foundation but diverge at the level of systemic immune organization, then disease progression should not be modeled as a single severity continuum [10,13]. Instead, it may be better represented by at least two partially distinct coordinates: one axis reflecting the shared inflammatory component of psoriatic disease and a second axis reflecting positional shift toward a PsA-associated systemic state.

To test this concept, we developed a cross-modality framework from four core public datasets spanning whole-blood DNA methylation, PBMC single-cell RNA sequencing summarized at the subject level, skin RNA sequencing, and purified CD4^+^ T-cell methylation, and used two additional public skin cohorts for external contextual checks. Using these complementary data layers, we defined two disease coordinates: a disease inflammatory response axis (DIR) and a contrast-resolved systemic state coordinate (CRS) representing the additional systemic component associated with PsA. We then examined whether these coordinates could distinguish shared psoriatic inflammatory burden from the additional systemic biology associated with PsA. Our results support a model in which PsA is distinguished from psoriasis by an additional systemic immune state axis rather than by increased skin inflammatory burden alone.

## 2. Results

### 2.1. Internal Resampling Supports Biological Coordinate Interpretation Rather than Clinical Classification

**Figure 1:**
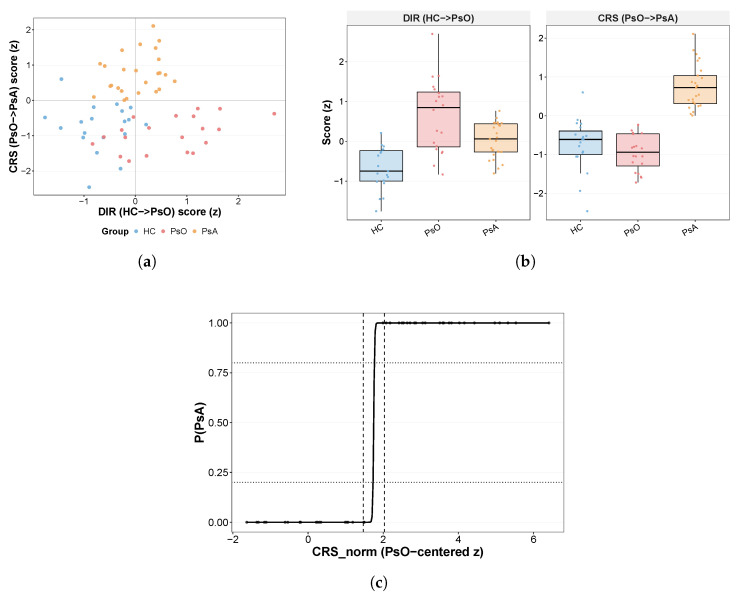
Discovery of DIR and CRS in whole-blood methylation. (**a**) Two-dimensional DIR–CRS map in GSE200376 showing the distribution of healthy controls (HCs), psoriasis without arthritis (PsO), and psoriatic arthritis (PsA) samples. (**b**) Group-wise DIR and CRS distributions demonstrate preferential separation of HC from PsO by DIR and of PsO from PsA by CRS. (**c**) Logistic modeling of normalized CRS defines a discovery-derived reference band used for downstream positioning of systemic states. Black points indicate observed class labels (PsO = 0, PsA = 1), the fitted curve shows predicted P(PsA), horizontal dotted lines mark the heuristic 0.20 and 0.80 probability cut points, and vertical dashed lines mark the corresponding $CRS_{\mathrm{norm}}$ lower and upper band limits used for downstream positioning. Together, these data are consistent with a model in which psoriatic disease contains both a shared inflammatory axis and an additional PsA-related systemic immune state axis.

We assessed the internal stability of the GSE200376 framework using leakage-aware resampling grouped by Sentrix ID where feasible. Internal discrimination was modest. The full-model area under the receiver operating characteristic curve (AUC) was 0.650 (95% CI, 0.474–0.826) for DIR and 0.682 (95% CI, 0.521–0.843) for CRS; the DIR confidence interval included the chance level of 0.5, and the CRS lower bound was only slightly above 0.5. Calibration was also imperfect, with calibration slopes of 0.504 for DIR and 0.430 for CRS (Supplementary Figure S1). These findings do not support interpretation of the discovery framework as a clinical prediction tool in its current form. Instead, they support its use as a biologically interpretable positional coordinate framework for organizing the systemic relationship among HC, PsO, and PsA (Figure 1). Consistent with this interpretation, logistic modeling of normalized CRS yielded a discovery-derived reference interval that was subsequently transferred as a positional reference to the independent single-cell cohort.

### 2.2. Subject-Level Single-Cell Profiles Show CRS-Intermediate Average Positioning Between Psoriasis and Psoriatic Arthritis

**Figure 2:**
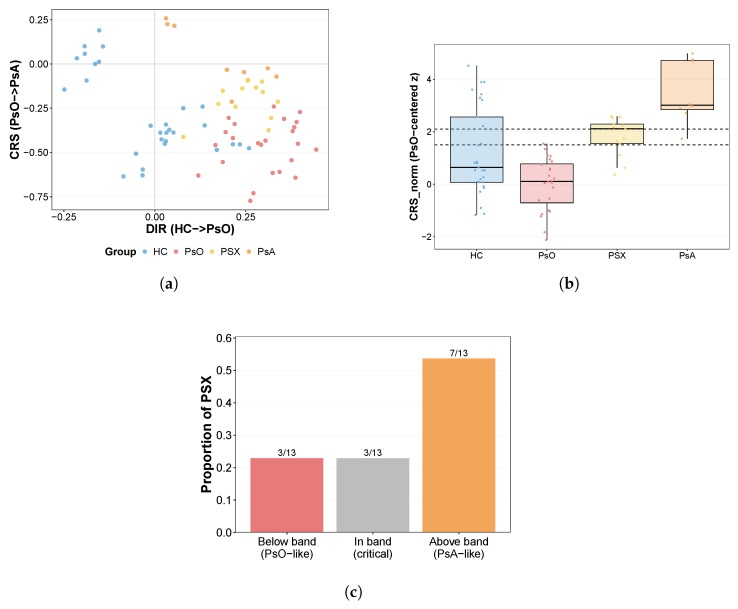
GSE194315 subject-level single-cell transcriptomic analysis recapitulates the systemic immune state coordinate while showing attenuation of the PSX above-band signal. (**a**) Overall DIR–CRS map across the untreated analysis set. (**b**) Discovery-aligned normalized CRS values separate PsA from PsO, with PSX showing heterogeneous intermediate positioning. Horizontal dashed lines indicate the lower and upper limits of the GSE200376-derived CRS reference band after PsO-centered normalization in GSE194315. (**c**) Among PSX subjects in the untreated analysis set, 7 of 13 were above-band, 3 of 13 were in-band, and 3 of 13 were below-band. This distribution is interpreted as symptom-subgroup heterogeneity rather than evidence of imminent arthritis conversion.

To determine whether the two-axis structure was recapitulated in an independent systemic cohort, we examined subject-level PBMC single-cell RNA sequencing data from GSE194315 using its untreated analysis set as the primary implementation (Figure 2). DIR again aligned more strongly with the shared psoriatic disease burden, whereas CRS separated PsA from PsO, with the source-defined PSX arthralgia subgroup showing intermediate average positioning but greater heterogeneity. At the overall subject level, the mean DIR score was −0.014 in HC, 0.301 in PsO, 0.250 in PSX, and 0.177 in PsA. In contrast, the mean CRS score increased from −0.465 in PsO to −0.203 in PSX and 0.039 in PsA.

The same overall pattern was retained in harmonized age- and sex-adjusted score-level checks, in which DIR remained higher in PsO than in HC and CRS remained higher in both PSX and PsA than in PsO (Supplementary Table S3).

Application of the discovery-derived CRS reference band clarified both the primary signal and its treatment sensitivity. In the primary GSE194315 analysis, no PsO subjects were positioned above the band (0/24 above-band; 23 below-band; and 1 in-band), whereas most PsA subjects were positioned above it (7/8 above-band). The source-defined PSX subgroup was heterogeneous: 7 of 13 subjects were above-band, 3 of 13 were in-band, and 3 of 13 were below-band. Within this cohort-specific signature learning framework, PsO versus PsA separation based on normalized CRS was complete, but this should be interpreted only as a small-sample internal anchoring check rather than as independent classification performance because the GSE194315 CRS signature was learned from PsO and PsA subjects within the same cohort. Compared with the all-available sensitivity analysis, the primary GSE194315 analysis attenuated PSX above-band occupancy, indicating that PSX above-band localization should not be treated as a stable standalone primary claim. The more robust non-training observation was therefore the heterogeneous PSX distribution together with the cell-type-level enrichment described below. Additional CRS localization panels are shown in Supplementary Figure S2. These findings support a systemic PsO-to-PsA coordinate structure in GSE194315 while emphasizing that the public metadata do not establish whether PSX joint pain reflects prodromal PsA or alternative musculoskeletal conditions.

Sensitivity analyses reinforced the need for this cautious interpretation. Applying the all-available score model after simply excluding treated subjects yielded a slightly higher PSX above-band count (8/13), whereas deriving the CRS signature and scores within the untreated analysis set yielded 7/13. Thus, excluding medication-annotated subjects only after score construction is not equivalent to defining the CRS signature from untreated PsO and untreated PsA subjects. The untreated-analysis-set workflow was therefore used for the main figures and text, while treatment sensitivity summaries are provided in Supplementary Table S4.

### 2.3. PSX-Enriched Above-Band Compartments and Multicompartment Pathway State Remodeling Localize CRS-Related Biology

**Figure 3:**
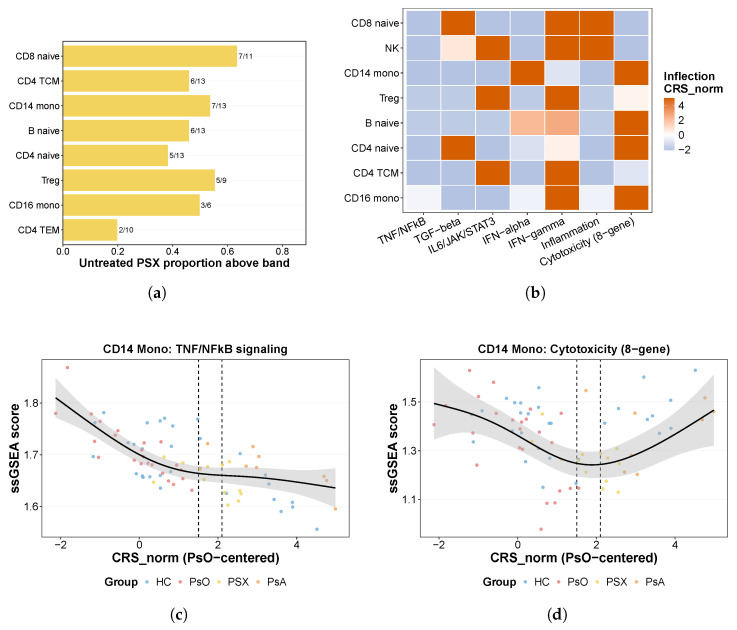
In the GSE194315 untreated analysis set, CRS localizes PSX-enriched above-band compartments and multicompartment pathway state remodeling associated with the PsA-related systemic state. (**a**) The fraction of PSX subjects occupying above-band states differs by cell type, highlighting PSX-enriched compartments such as CD8 naive T-cells, CD4 central memory T-cells, CD14 monocytes, B naive cells, CD4 naive T-cells, Treg cells, and CD16 monocytes. (**b**) Focused pathway inflection mapping for selected Hallmark programs plus the 8-gene cytotoxicity signature summarizes descriptive pathway-position patterns along the CRS continuum across multiple immune compartments. In panel (**b**), color denotes the estimated CRS_norm inflection position rather than pathway activity magnitude, with warmer colors indicating inflection at higher, more PsA-aligned CRS positions. (**c**) A representative CD14 monocyte TNF/NFκB signaling curve shows inflammatory pathway state remodeling across the CRS continuum. (**d**) A representative CD14 monocyte 8-gene cytotoxicity signature curve illustrates high-CRS cytotoxicity signature activity across the same systemic CRS continuum. In panels (**c**,**d**), vertical dashed lines mark the lower and upper limits of the GSE200376-derived CRS reference band after GSE194315 PsO-centered normalization; HC points are shown for visual context only, and black smooths show pooled PsO/PSX/PsA CRS continuum GAM fits.

We examined whether the CRS-related signal was uniformly distributed across immune compartments or preferentially localized to specific cell types. In contrast to the attenuated overall PSX above-band count, PSX-enriched above-band analysis remained more informative at the cell-type level (Figure 3). The strongest PSX-enriched above-band compartments included CD8 naive T-cells, CD4 central memory T-cells, CD14 monocytes, and B naive cells. For example, no PsO subjects (0/18) occupied the above-band state in CD8 naive T-cells, whereas 7 of 11 PSX subjects did so (Fisher FDR $=2.54\times 10^{-3}$; permutation FDR $=5.60\times 10^{-3}$). Similar FDR-significant patterns were observed for CD4 central memory T-cells (0/24 vs. 6/13 PSX above-band), CD14 monocytes (1/24 vs. 7/13), B naive cells (0/20 vs. 6/13), CD4 naive T-cells (0/24 vs. 5/13), Treg cells (0/10 vs. 5/9), and CD16 monocytes (0/15 vs. 3/6).

Because several of these PsO versus PSX contingency tables contained zero above-band PsO observations, we additionally computed continuity-corrected effect-size summaries. Across the seven FDR-significant PSX-enriched compartments, Haldane–Anscombe odds ratios ranged from 18.1 to 61.7 and bounded risk differences ranged from 0.385 to 0.636; representative large effects were observed for CD8 naive T-cells, CD4 central memory T-cells, B naive cells, and Treg cells (Supplementary Table S5).

We then examined whether CRS reflected changes in broad cell abundance or changes in cell-intrinsic transcriptional state. Abundance-focused summaries did not suggest that the CRS signal was explained solely by redistribution of major blood cell populations. Instead, the CRS-related signal was more clearly localized in within-compartment above-band enrichment and pathway state modeling. Additional cell-type abundance and pathway detail panels, including representative CD16 monocyte TNF/NFκB and 8-gene cytotoxicity signature trajectories plotted with the same pooled-smooth convention as the main figure, are shown in Supplementary Figure S3; treatment sensitivity summaries are provided in Supplementary Table S4.

Pathway modeling along the normalized CRS continuum identified 72 inflection events across eight selected cell types: CD8 naive T-cells, NK cells, CD14 monocytes, Treg cells, B naive cells, CD4 naive T-cells, CD4 central memory T-cells, and CD16 monocytes. Of these, 49 inflection events were downward and 23 were upward. Recurrent pathway changes involved TNF/NFκB signaling, TGF-β signaling, IL6/JAK/STAT3 signaling, inflammatory response, interferon response programs, and 8-gene cytotoxicity signature activity. In Figure 3c,d, representative CD14 monocyte TNF/NFκB and 8-gene cytotoxicity signature curves illustrate inflammatory and high-CRS cytotoxicity along the CRS continuum; the colored points show individual subject-level pathway scores, while the black smooths summarize pooled PsO/PSX/PsA trajectories. These patterns were not consistent with a uniform increase in inflammatory signaling across all compartments. Together, these summaries localized CRS-related pathway remodeling to innate, regulatory, naive-memory lymphoid, B-cell, and monocyte compartments. A broader exploratory all-cell-type screen in the untreated analysis set identified 144 estimable pathway–cell type summaries across 17 eligible cell types, of which 16 yielded at least one estimable maximal-change summary, and is retained as supplementary context (Supplementary Figure S7 and Table S6).

The inferential support for this interpretation was derived from the PSX-enriched above-band enrichment tests, whereas the inflection heatmap and representative pathway curves provided descriptive summaries of where along the CRS continuum these transcriptional changes were concentrated. Additional robustness checks supported the directional stability of these summaries relative to the all-available analysis. Across 72 pathway–cell type pairs, 60 retained the same Spearman direction and 65 retained the same GAM slope direction compared with the all-available outputs (Supplementary Table S4). These results indicate that the pathway summaries were more stable at the level of directional localization than at the level of exact inflection point placement.

### 2.4. Skin RNA Sequencing Captures Inflammatory Tissue Burden and Shows Limited Additional PsA-Related Separation

**Figure 4:**
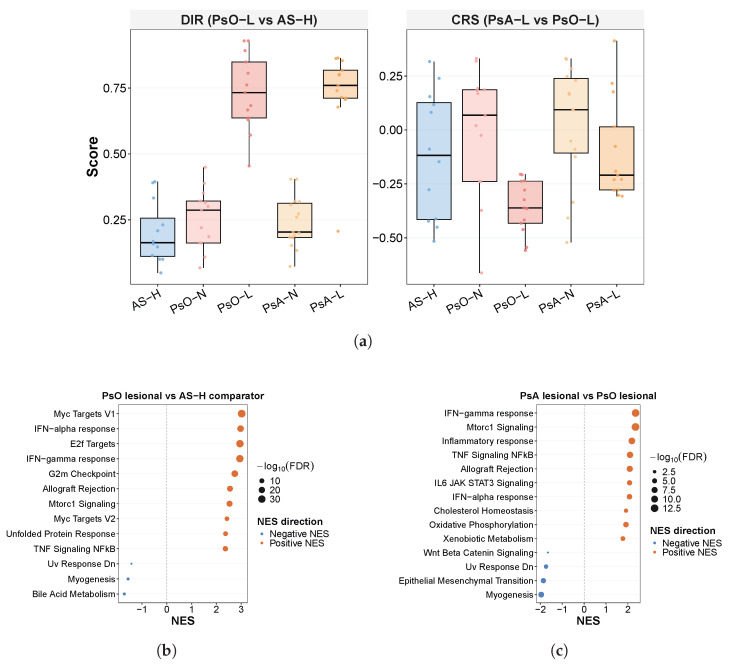
Skin transcriptomic profiles primarily reflect shared inflammatory burden and show limited additional PsA-related separation within the same tissue state. (**a**) DIR distributions show strong lesional inflammatory state separation, whereas CRS shows more limited additional PsA-related separation within lesional skin. In this dataset, AS-H denotes healthy-appearing skin from ankylosing spondylitis comparators rather than skin from unaffected healthy volunteers. (**b**) Hallmark enrichment for psoriatic lesional skin versus the dataset-specific non-psoriatic comparator highlights robust inflammatory tissue activation. (**c**) Hallmark enrichment for PsA lesional skin versus PsO lesional skin shows that, despite minimal additional separation at the individual-gene level, ranked-pathway-level differences remain detectable within the lesional state.

We examined whether the psoriasis-to-PsA distinction could be explained primarily at the level of skin inflammatory burden. In GSE186063, lesional skin transcriptomes strongly separated inflammatory skin states from the healthy-appearing ankylosing spondylitis comparator skin (AS-H) and generated a robust lesion-driven differential expression burden (Figure 4). PsO lesional skin versus the comparator AS-H state yielded 6619 FDR-significant genes, and PsA lesional skin versus the comparator AS-H state yielded 8019 FDR-significant genes. Within-disease lesional versus non-lesional contrasts were similarly strong (6415 FDR-significant genes in PsO and 8861 in PsA).

In contrast, psoriasis versus PsA comparisons within the same tissue state showed minimal additional separation at the individual-gene level. PsA lesional skin versus PsO lesional skin yielded no FDR-significant genes, and PsA non-lesional skin versus PsO non-lesional skin yielded only one FDR-significant feature, KRT16P3 (ENSG00000214822). Ranked Hallmark enrichment analyses refined this interpretation further. Relative to the robust lesional versus AS-H contrasts, psoriasis versus PsA differences within the same tissue state were more evident at the pathway level than at the level of individually significant genes. In the lesional comparison, interferon response, inflammatory response, TNF/NFκB, and IL6/JAK/STAT3 programs remained detectably enriched in PsA relative to PsO (Figure 4). Harmonized age- and sex-adjusted score-level checks sharpened this interpretation further, showing strong DIR_skin_ separation for PsO-L versus AS-H, no additional DIR_skin_ separation for PsA-L versus PsO-L, and a modest but significant CRS_skin_ shift for PsA-L versus PsO-L (Supplementary Table S3). Together, these results indicate that skin transcriptomes primarily capture the shared inflammatory burden of psoriatic disease, while only limited additional PsA-related separation remains detectable within the same tissue state and is unlikely to account fully for the stronger systemic distinction observed in blood-derived datasets. Because the official skin CRS_skin_ score required a ranked-signature fallback rather than a stable core lesional signature, this residual CRS_skin_ signal should be interpreted cautiously.

External skin checks further supported this interpretation. In the small balanced PsA skin bulk RNA-seq cohort GSE205748, exact permutation rank-sum tests showed reproducible lesional enrichment of IL6/JAK/STAT3, inflammatory response, interferon-alpha, interferon-gamma, and TNF/NFκB programs relative to both HC skin and uninvolved PsA skin (all FDR $\leq 9.87\times 10^{-5}$), whereas TGF-β signaling remained weak (Supplementary Figure S5). Because GSE205748 did not include psoriasis without arthritis, these data corroborated lesion-associated PsA skin inflammation but did not directly test psoriasis versus PsA skin separation. In the external spatial skin cohort GSE202011, projected DIR_skin_ scores again separated lesional PsO and lesional PsA from HC skin (both FDR $=1.75\times 10^{-3}$) without additional PsA versus PsO lesional separation (FDR =0.547), whereas projected CRS_skin_ scores showed only modest lesion versus HC shifts (FDR $=2.62\times 10^{-2}$ for PsO-L versus HC; FDR $=2.21\times 10^{-2}$ for PsA-L versus HC) and no lesional PsA versus PsO separation (FDR =0.902; Supplementary Figure S6). This external non-replication of lesional CRS_skin_ separation is consistent with instability introduced by the ranked-signature fallback used for the official skin CRS_skin_ score.

### 2.5. Purified CD4^+^ T-Cell Methylation as an Exploratory Within-Cohort Analysis

Finally, we examined purified CD4^+^ T-cell methylation as a small exploratory layer. CpG-level differential methylation support was limited in this cohort: the PsO versus HC contrast yielded one FDR-significant CpG (cg17864916), whereas the PsA versus PsO contrast yielded none. Using fallback top-|Δβ| CpG sets, exploratory within-cohort score construction nevertheless produced baseline-referenced directional shifts broadly compatible with the disease axis framework. The corresponding DIR–CRS map and group-wise score distributions are shown in Supplementary Figure S4. We therefore interpret GSE236694 only as a small exploratory within-cohort analysis.

## 3. Discussion

The main contribution of this study is not to argue that psoriasis is systemic, which is already well supported, but to define which additional systemic features distinguish psoriasis without arthritis from psoriatic arthritis [1,2,3]. Across the analyzed datasets, the psoriasis–PsA spectrum was better represented by at least two partially distinct coordinates: a shared disease inflammatory response axis (DIR) and an additional CRS coordinate that captured the systemic component most closely aligned with PsA. In this framework, PsA is not simply a more severe version of psoriasis. Rather, it is associated with an added systemic immune state component superimposed on a shared inflammatory background [10,11,13].

This framing also clarifies the novelty of the study. The goal was not to claim a new fixed biomarker panel from any single cohort, nor to replace earlier single-layer analyses that identified cell-type markers, machine learning classifiers, or purified cell methylation differences [5,7]. Instead, the advance here lies in organizing those disparate signals into a single cross-modality coordinate structure that separates shared psoriatic inflammatory burden from the additional systemic state associated with PsA and then testing where that structure reproduces, weakens, or fails across blood- and skin-derived layers. Importantly, this cross-modality interpretation does not assume feature-level equivalence between methylation-derived and transcriptome-derived signals; instead, it tests whether independently defined PsO versus PsA contrasts preserve comparable relative positioning after PsO-centered normalization.

Several findings support this interpretation. In the discovery blood methylation cohort, DIR and CRS were only weakly correlated, arguing against a single-axis severity model. DIR primarily tracked the shift from HC to PsO, whereas CRS primarily tracked the shift from PsO to PsA. In the independent PBMC single-cell cohort, the primary GSE194315 reanalysis preserved PsO versus PsA separation but attenuated the PSX above-band signal, indicating that PSX positioning is heterogeneous and sensitive to whether treated PsA subjects are included during signature learning. Together, these observations support an added systemic immune state dimension while arguing against overinterpreting subject-level PSX placement from this cross-sectional public cohort [11,12,14].

An additional strength of the current framework is that it localizes the CRS-related signal to specific immune compartments. In the primary GSE194315 reanalysis, PSX-enriched above-band compartments remained detectable in naive and central-memory T-cell, monocyte, B-cell, and regulatory compartments. More broadly, the GSE194315 results indicate that the psoriasis-to-PsA distinction is represented more strongly by coordinated multicompartment pathway state remodeling than by a simple redistribution of major blood cell populations [8,14].

The descriptive pathway analyses refine this interpretation further. CRS was associated with patterns consistent with structured pathway state remodeling across TNF/NFκB, TGF-β, IL6/JAK/STAT3, inflammatory response, interferon response, and 8-gene cytotoxicity signature activity. These changes did not resemble uniform hyperinflammation. Instead, different pathways changed in different directions across different cell types, supporting the interpretation that PsA involves a qualitatively different systemic immune configuration [13,15]. The representative monocyte trajectories illustrate this point at the compartment level. CD14 and CD16 monocyte programs showed CRS-positioned changes in TNF/NFκB signaling and 8-gene cytotoxicity signature activity, consistent with involvement of altered myeloid inflammatory organization rather than a uniform increase in all inflammatory pathways. These monocyte-centered patterns, including the 8-gene cytotoxicity signature results, should be interpreted as transcriptional pathway state associations rather than as direct evidence of altered monocyte effector function or functional cytotoxic capacity. This distinction matters because it suggests that the central biology of the psoriasis-to-PsA distinction lies in altered immune organization, not merely in greater magnitude of inflammation.

The skin RNA-seq results further sharpen the biological interpretation of the two-axis model. Lesional skin robustly reflected inflammatory burden, as expected for active psoriatic disease [6,9], whereas psoriasis versus PsA comparisons within the same tissue state showed minimal additional separation at the individual-gene level and only more modest ranked pathway-level differences. This does not diminish the importance of skin inflammation in psoriatic disease. Rather, it suggests that skin inflammation corresponds more closely to the shared disease component represented by DIR, whereas the systemic distinction between psoriasis and PsA is better captured by blood-derived molecular and cellular states. In practical terms, this means that the psoriasis versus PsA distinction cannot be adequately explained by cutaneous inflammatory burden alone.

The external skin analyses were informative precisely because they tested different pieces of the skin argument. GSE205748 reinforced that lesional PsA skin reproducibly carries strong inflammatory pathway activity relative to healthy and uninvolved skin, but it could not address psoriasis versus PsA skin separation because psoriasis without arthritis was not represented in that cohort. GSE202011 addressed that narrower question more directly at the sample level and reproduced lesion versus healthy inflammatory burden without reproducing additional lesional CRS_skin_ separation between PsO and PsA. Taken together, these external checks support DIR_skin_ as a lesion burden summary more strongly than they support CRS_skin_ as a stable external skin marker of PsA-specific separation.

The present study also clarifies how the CRS reference band should be interpreted. The band derived from GSE200376 was useful for positioning subjects along a PsO-like to PsA-like continuum, but it should not be treated as a clinical threshold, a deployable diagnostic rule, or an individual prognostic biomarker. The discovery methylation model showed only modest internal discrimination and imperfect calibration, and the band itself was derived from cross-sectional data. Accordingly, the framework is best understood as a biological coordinate system for organizing systemic disease states rather than as a ready-to-use clinical classifier.

Several limitations should be acknowledged. First, all analyses were performed on public datasets, so the study necessarily inherits the cohort composition, annotation quality, inclusion and exclusion criteria, and technical heterogeneity of the original studies (see Supplementary Table S2 for source cohort eligibility notes). Second, the available cohorts were modest in size, especially for the untreated PsA subset of GSE194315, the purified CD4^+^ T-cell methylation dataset, and the external skin checks. Third, the data are cross-sectional and do not directly establish temporal ordering, disease staging, prognosis, or causality; where dynamic language is used in this manuscript, it refers to inferred biological positioning rather than observed longitudinal movement.

Fourth, key clinical variables were incomplete or unavailable across datasets (Supplementary Table S2). Treatment status, disease duration, age at disease onset, detailed symptom duration, PASI, and PsA disease activity measures could not be harmonized across cohorts. In GSE194315, systemic medication use was recorded only at the drug name level and was concentrated in PsA subjects. We therefore defined the primary GSE194315 analysis by excluding subjects with recorded systemic medication before signature learning, scoring, and downstream modeling, but this reduced the PsA learning set to eight untreated subjects and could not account for medication dose, duration, washout, treatment timing, treatment response, or baseline disease severity before treatment. In GSE186063, the source article reported substantial recent group-level treatment history, including biologic exposure, but sample-level treatment covariates were not available in GEO and could not be adjusted in the skin transcriptomic contrasts. Fifth, subject-level ethnicity was available for GSE194315 and cohort-level patient ethnicity was reported for GSE236694, but ancestry/ethnicity was not consistently reported or harmonizable across datasets, limiting assessment of ancestry-related generalizability.

Sixth, the exact GSE200376-derived whole-blood methylation score model was not externally validated in an independent whole-blood methylation cohort. The current study therefore supports cross-modality consistency of the framework more strongly than transportability of a fixed same-modality methylation predictor. Seventh, the GSE194315 PSX subgroup was defined retrospectively from source metadata as psoriasis with joint pain but without CASPAR-classified PsA, and the public records did not adjudicate alternative causes of musculoskeletal pain. The PSX findings should therefore be interpreted as enrichment within a symptom-defined subgroup rather than as proof of imminent PsA conversion. This cautious framing is consistent with the 2023 EULAR points to consider, which treat arthralgia in people with psoriasis as a risk context while emphasizing the need to consider alternative diagnoses [16]. Eighth, the discovery and comparison layers do not all share the same assay or tissue context, which limits any claim of a single transferable biomarker panel. Ninth, the purified CD4^+^ T-cell methylation cohort was small, had limited CpG-level DMP support, and used the same cohort for exploratory score construction and evaluation; those findings should therefore be interpreted only as exploratory within-cohort observations.

Despite these limitations, convergent evidence from whole-blood methylation, subject-level PBMC single-cell transcriptomics, skin RNA sequencing, and exploratory purified CD4^+^ T-cell methylation supports the biological utility of the proposed framework. More broadly, these findings are consistent with a model in which psoriasis and PsA share a systemic inflammatory backbone but diverge through an additional multicompartment immune state axis. Future studies should test this model in longitudinal cohorts, determine whether CRS-like profiles can be detected before clinically overt arthritis and how early they emerge, assess whether CRS differs across PsA clinical domains such as peripheral arthritis, enthesitis, dactylitis, and axial involvement, and define how metabolic, microbial, and immune network perturbations converge on the systemic state difference identified here.

## 4. Materials and Methods

### 4.1. Study Design and Public Datasets

This study used four core publicly available transcriptomic and epigenomic datasets, representing complementary biological layers of psoriatic disease within a cross-modality disease axis framework (Supplementary Table S1). GSE200376, a peripheral blood DNA methylation cohort containing healthy controls (HCs), psoriasis without arthritis (PsO), and psoriatic arthritis (PsA), was used as the primary systemic discovery dataset to define the disease coordinates [17,18]. GSE194315, a subject-level peripheral blood mononuclear cell (PBMC) single-cell RNA sequencing dataset with surface epitope profiling, was used as an independent systemic transcriptomic comparison cohort for subject-level positioning, PSX positioning analysis, PSX-enriched above-band compartment analysis, and pathway state remodeling. The primary GSE194315 analysis excluded subjects with recorded systemic medication; all-available analyses were summarized only as treatment sensitivity context in Supplementary Table S4 and were not used for the primary results [5,19]. GSE186063, a skin RNA sequencing dataset with lesional and non-lesional biopsies plus healthy-appearing skin from ankylosing spondylitis comparators, was used as the cutaneous anchoring cohort to determine whether the psoriasis versus PsA distinction could be explained by skin inflammatory burden [6,20]. GSE236694, a small exploratory purified circulating CD4^+^ T-cell DNA methylation cohort, was included only to examine whether disease axis structure might also be detectable in a more cell-enriched methylation context [7,21]. Two additional public skin cohorts, GSE205748 and GSE202011, were used later as external contextual checks for the skin-layer interpretation [22,23,24,25].

All analyses were performed on publicly available, de-identified data. No new human participants were recruited for this study. Human participant oversight and consent procedures were handled by the original source studies according to local requirements; source-site and ethics/consent details that could be traced from the corresponding publications or GEO records are summarized in Supplementary Table S2. For the present secondary analysis, no identifiable participant-level information was accessed. Additional notes on how DIR and CRS were operationalized and interpreted across modalities are summarized in Appendix Table A1.

### 4.2. Metadata Harmonization and Analysis Cohort Definitions

Sample- and subject-level metadata were harmonized across datasets using the integrated project metadata tables and dataset-specific design tables generated during preprocessing. Disease groups were standardized to HC, PsO, PsA, and, where available, PSX. Clinical diagnoses, disease group labels, and source cohort eligibility definitions were taken from the original studies and were not clinically re-adjudicated in the present analysis. Analysis-specific filtering was then applied for metadata harmonization, assay-level quality control, the GSE194315 systemic medication exclusion used for the primary analysis, and prespecified cell count thresholds. Dataset-specific source-site and eligibility notes are summarized in Supplementary Table S2. In GSE194315, the source study defined PSX as the subgroup of psoriasis subjects reporting joint pain but not meeting CASPAR classification criteria for PsA [5,26]. We therefore treated PSX here as a dataset-defined arthralgia subgroup rather than as a newly assigned clinical label. Because the public source records did not adjudicate alternative causes of joint pain, the PSX label in the current study should not be interpreted as proof of prodromal PsA. The PsA label in the public datasets was retained as provided by the source studies; detailed musculoskeletal domain annotations such as peripheral arthritis versus enthesitis, dactylitis, axial involvement, swollen/tender joint counts, and composite PsA disease activity indices were not available in harmonized form and therefore could not be modeled separately. For GSE186063, skin state was retained separately as healthy-appearing ankylosing spondylitis comparator skin (AS-H), non-lesional (N), or lesional (L), and the analysis unit was defined as AS-H, PsO-N, PsO-L, PsA-N, or PsA-L. Accordingly, AS-H in this dataset denotes a within-dataset non-psoriatic skin comparator rather than skin from unaffected healthy volunteers. For GSE194315, the subject rather than the individual cell was treated as the primary statistical unit. Subject-level metadata were linked to cell-level metadata by subject identifier. A prespecified threshold of 100 retained cells per subject was used as a basic quality control safeguard to avoid unstable subject-level cell-type proportion estimates and pseudobulk analyses in very low-coverage subjects. No subject in the final primary cohort fell below this threshold. A disease stage factor was prespecified in the fixed order HC → PsO → PSX → PsA. For ordered trend analyses, this stage order was encoded as an ordinal numeric variable (HC = 0, PsO = 1, PSX = 2, and PsA = 3) to test monotonic associations of cell-type proportions and signature scores with disease stage rank. This coding was used only to represent rank order and was not intended to imply equal biological spacing or observed longitudinal progression between groups.

Clinical annotation depth differed substantially across datasets. Age and sex were available for the four core datasets and were used in harmonized score-level sensitivity checks where applicable. For GSE200376, the GEO sample-level treatment protocol field was annotated as “no” for all samples, but this was interpreted as a sample-processing/protocol annotation rather than as patient-level evidence of untreated clinical status. Because individual medication histories, washout criteria, treatment duration, and dose intensity were not available in the harmonized public metadata or accessible source records, treated and untreated subjects could not be distinguished or analyzed separately in this cohort [17,18]. For GSE236694, the source article reported that patients were not receiving relevant systemic immunomodulating therapy at study inclusion after prespecified washout periods for conventional systemic therapies or biologic drugs [7]. In GSE194315, systemic medication information was available at the drug name level. PsO subjects were annotated as not receiving systemic medication, whereas medication use was concentrated in PsA subjects. For the primary GSE194315 analysis, subjects with recorded systemic medications were excluded from the primary analysis set before signature learning, scoring, band occupancy analysis, and pathway modeling, leaving 29 HC, 24 PsO, 13 PSX, and 8 PsA subjects. The excluded subjects consisted of one guselkumab-treated PSX subject and 20 medication-annotated PsA subjects. Because only drug names, not dose, duration, washout, treatment timing, or treatment response, were available, the all-available dataset was used only for treatment sensitivity context (Supplementary Table S4), not for primary GSE194315 results. PASI was available only for subsets of GSE194315 and GSE236694 and was absent from the whole-blood discovery methylation cohort GSE200376 and the skin RNA-seq cohort GSE186063. Disease duration, age at disease onset, symptom duration, and detailed treatment duration or dose intensity were not available in the public records in a harmonized form. Ethnicity was available at the subject level for GSE194315, which included White, Asian, Hispanic, African American, Other, and unreported categories, and the GSE236694 source article reported that all patients were of White European ethnicity [7]; however, sample-level ancestry fields were not available in GEO or the harmonized metadata for GSE236694, and ethnicity was not consistently available across the remaining core datasets. These metadata constraints were treated as limitations of the public data design rather than as analyzable covariates in all datasets.

Across the four core datasets, group standardization and subject-level alignment were applied during metadata harmonization, and harmonized age- and sex-adjusted score-level consistency checks were performed. Dataset characteristics are summarized in Supplementary Table S1, clinical metadata availability is summarized in Supplementary Table S2, and harmonized age- and sex-adjusted score-level consistency checks are summarized in Supplementary Table S3.

### 4.3. Preprocessing of DNA Methylation Datasets

Raw Illumina methylation IDAT files were processed in R using minfi (v1.56.0) [27]. For both GSE200376 and GSE236694, raw Illumina methylation IDAT basenames were matched to dataset-specific target tables keyed to GEO sample identifiers (GSM) to define the analysis cohorts. Detection *p* values were computed with minfi::detectionP, and per-sample quality control metrics, including call rate and median methylated and unmethylated signal intensities, were recorded. Background correction and normalization were performed using preprocessNoob [28]. Beta values and M values were extracted with getBeta and getM, respectively.

For the primary GSE200376 differential methylation analysis, probes were retained only if detection p≤0.01 in at least 95% of samples, after which non-CpG probes, sex chromosome probes, and published cross-reactive and SNP-overlapping probes were excluded using the EPIC blacklist files provided as Supplementary Tables S1 and S4–S6 in the original annotation resource by Zhou et al. [29].

For GSE236694, the primary differential methylation analysis likewise retained probes with detection p≤0.01 in at least 95% of samples and then restricted the analysis to autosomal probes. Sample-level quality control metrics were reviewed for both methylation cohorts, and this review did not identify any sample requiring additional exclusion in the primary differential methylation analyses. Filtered beta matrices, M value matrices, detection *p* matrices, and per-sample quality control tables were retained as downstream analysis inputs.

Because GSE200376 served as the primary whole-blood discovery methylation cohort, a stringent blacklist-based probe filter was applied, whereas GSE236694 was treated as a small exploratory purified CD4^+^ T-cell cohort and therefore used a minimal probe filter limited to detection-passing autosomal probes in order to preserve analyzable probe coverage.

### 4.4. Construction of the GSE200376 Discovery Coordinate System

GSE200376 was used to construct the primary discovery coordinate system. Two axes were defined. The disease inflammatory response axis (DIR) was anchored to the PsO versus HC contrast and was intended to capture the shared inflammatory component of psoriatic disease. The contrast-resolved systemic state coordinate (CRS) was anchored to the PsA versus PsO contrast and was intended to capture the additional systemic state associated with PsA relative to PsO.

Differentially methylated probes (DMPs) were identified from *M* values using limma (v3.66.0), and differentially methylated regions (DMRs) were identified using DMRcate (v3.6.0) [30,31]. For pairwise comparisons, the primary total signal model was specified as

∼0+group+age+sex+sentrix_id,

where group denotes the harmonized disease group factor (HC, PsO, or PsA) and sentrix_id denotes the Illumina BeadChip/Sentrix slide identifier derived from the IDAT basename. A cell-adjusted sensitivity model additionally included the estimated whole-blood cell fractions CD8T, CD4T, NK, Bcell, and Mono. Ordered trend analyses across HC, PsO, and PsA used a numeric trend term coded as HC =0, PsO =1, and PsA =2. This coding was used only to represent ordered disease stage rank for trend testing and was not intended to imply equal biological spacing or observed longitudinal progression. The total signal trend model was specified as

∼trend+age+sex+sentrix_id

and a cell-adjusted sensitivity trend model additionally included the same whole-blood cell fraction covariates. Statistical testing was performed on *M* values, and strict DMPs were defined as probes with Benjamini–Hochberg false discovery rate (FDR) <0.05 and |Δβ|≥0.02. DMRs were called using a strict seed threshold of FDR <0.05 in the reported primary run.

Whole-blood cell fractions were estimated with the FlowSorted.Blood.EPIC reference-based deconvolution workflow using estimateCellCounts2 with the preprocessNoob processing method and IDOL probe selection [32]. Sample identifiers were harmonized to GSM IDs before merging estimated cell fractions with the design table, and cell fraction row sums were checked and renormalized when minor numerical drift from 1.0 was detected.

Final GSE200376 DIR and CRS scores were computed from CpG signatures derived from the internal five-fold resampling procedure used for score construction. Within each training fold, CpGs were selected de novo from the relevant limma contrast, and probes selected in at least three valid folds, defined here as folds in which the training contrast was estimable and a fold-specific score model could be fit, were retained for final scoring; this three-fold rule was used as a heuristic stability filter rather than as a formal optimality criterion. This yielded 25 CpGs for DIR and 45 CpGs for CRS in the final scoring set, reflecting axis-specific fold stability rather than a matched target feature count.

For each sample *j*, the raw score was computed as

$${\mathrm{Score}}_{j}^{\mathrm{raw}}=\sum_{i}w_{i}z\left(M_{ij}\right),$$

where $w_{i}$ is the limma moderated *t* statistic for CpG *i* from the relevant discovery contrast, and $z(M_{ij})$ denotes CpG-wise standardization of the sample *M* value using the mean and standard deviation of that CpG within the relevant reference set. For each axis, the *M* value of each retained CpG was first standardized using the mean and standard deviation of that CpG within the relevant reference groups (DIR: HC + PsO; CRS: PsO + PsA). These standardized CpG values were then combined as a weighted sum to obtain the raw score. The raw score was subsequently centered and scaled using the mean and standard deviation of the raw-score distribution within the same reference set, and the resulting reference-centered standardized scores were saved as the final DIR and CRS values used for downstream positioning and visualization. These axis-specific reference sets were chosen to preserve contrast-specific interpretability rather than to place DIR and CRS on a single shared assay-level scale.

### 4.5. Internal Stability and Calibration Assessment in GSE200376

The GSE200376 discovery framework was internally evaluated using leakage-aware nested resampling, but this procedure was used only to characterize the stability of the coordinate system and the discrimination and calibration of the derived score-based prediction models; it was not treated as external validation. Five-fold outer cross-validation was performed. Fold assignment was grouped by sentrix_id whenever feasible to reduce batch leakage; when grouped splitting was infeasible, a stratified fallback preserving class balance was used.

Within each training fold, feature selection was performed de novo using limma, and the weighted score was reconstructed using training data only. Fold-specific score standardization was based exclusively on the training mean and standard deviation. For each axis, prediction models were then fitted for the corresponding discovery contrast (DIR: HC vs. PsO; CRS: PsO vs. PsA) using ridge logistic regression (glmnet, v4.1-10), with a generalized linear model fallback implemented as a safeguard when penalized fitting was infeasible in a given fold [33]. The ridge penalty parameter was selected within each training fold by inner cross-validation using the 1-SE rule, defined here as the largest penalty with cross-validated AUC within one standard error of the maximum [34]. The full prediction model included the fold-specific standardized score, age, sex, and the first two principal components of the cell fraction matrix.

The area under the receiver operating characteristic curve (AUC), Brier score, calibration intercept, and calibration slope were recorded together with five-bin calibration tables and fold-level diagnostics.

### 4.6. Subject-Level and Cell-Type-Level DIR/CRS Scoring in GSE194315

GSE194315 was analyzed at the subject level using the untreated analysis set defined above. Subject-level cell-type proportions were first calculated from the filtered cell metadata. Pseudobulk RNA count matrices were then generated for each subject and annotated cell type by summing pre-normalization single-cell counts within each subject–cell type combination. Subject–cell type combinations represented by fewer than 30 retained cells were excluded from pseudobulk generation. Per-cell-type pseudobulk matrices and matching subject-level metadata tables were then constructed for downstream scoring and pathway analysis.

Cell-type-specific DIR and CRS signatures were derived independently within GSE194315 from pseudobulk contrasts using edgeR (v4.8.2), and signature scores were subsequently computed by single-sample gene set enrichment analysis using GSVA (v2.4.4) [35,36]. For each cell type, a signed ranking statistic was defined as

$$\mathrm{signed}\_\mathrm{stat}=\mathrm{sign}\left(\log FC\right)\sqrt{F},$$

where *F* is the quasi-likelihood test statistic from glmQLFTest [37]. This quantity was used only to rank genes for signature construction and was not interpreted as a formal inferential effect size estimate.

DIR signatures were learned using only HC and PsO subjects, whereas CRS signatures were learned using only PsO and PsA subjects; PSX subjects were explicitly excluded from signature learning and were scored only after the signatures had been fixed. For each contrast and cell type, signature learning required at least five subjects in each contrast group. The top 100 genes with the most positive signed statistic and the top 100 genes with the most negative signed statistic were retained as the UP and DOWN components of the signature, respectively. Per-cell-type scoring was then performed only when at least five genes remained in both the UP and DOWN components after matching to the expression matrix.

Per-cell-type scores were computed by ssGSEA using GSVA, with kcdf = “Gaussian”, absRanking = TRUE, and ssgsea.norm = TRUE. The final score was defined as

Score=ssGSEA(UP)−ssGSEA(DOWN).

Subject-level overall DIR and CRS scores were then computed as weighted means across cell types, using weights proportional to $\sqrt{n_{\mathrm{cells}}}$ when subject–cell type cell counts were available and equal weighting otherwise. In the final primary GSE194315 analysis, cell count weights were available for all retained subject–cell type combinations, so the equal weight fallback was not used. This $\sqrt{n_{\mathrm{cells}}}$ weighting scheme was used as a heuristic compromise between equal weighting and direct cell count weighting.

### 4.7. Definition of the CRS Reference Band and PSX-Enriched Above-Band Compartments

To formalize a reference interval along the systemic CRS continuum, CRS scores in GSE200376 were first normalized relative to the PsO distribution:

$$CRS_{\mathrm{norm}}=\frac{CRS-\mu_{\mathrm{PsO}}}{\sigma_{\mathrm{PsO}}}.$$

A logistic model for $P(\mathrm{PsA}|CRS_{\mathrm{norm}})$ was then fitted using the PsO and PsA samples only. The initial lower and upper band limits were defined as the normalized CRS values corresponding to predicted probabilities of 0.20 and 0.80, respectively. To summarize uncertainty and obtain the final applied band, class-balanced bootstrap resampling (1000 iterations) was performed, and the bootstrap medians of the lower and upper bounds were retained as the final CRS reference band. These probability cut points were used as a heuristic reference interval for the central reference zone rather than as optimized diagnostic thresholds.

Band stability in the discovery cohort was further assessed by repeated stratified five-fold cross-validation (50 repeats), in which CRS normalization and logistic fitting were repeated within each training split, and the resulting band limits were summarized across repeats. Concise summaries of the bootstrap and repeated-CV band stability analyses are reported in Supplementary Figure S1.

The GSE200376-derived band was then applied to the GSE194315 untreated analysis set after recalculating the PsO mean and standard deviation within the scRNA-seq cohort. For subject-level overall scores, normalization used the overall untreated PsO distribution, whereas for subject–cell type scores, normalization was performed separately within each cell type using the corresponding untreated PsO distribution. This transfer was based on contrast-level rather than feature-level equivalence: in both datasets, CRS was defined independently from the same PsO versus PsA biological contrast within the assay-specific data layer. PsO-centered normalization therefore aligned each cohort to its own internal psoriasis reference state, so that transferred band labels reflected relative displacement away from PsO rather than equality of methylation-derived and transcript-derived measurement scales. The transfer was interpreted only as a test of whether a discovery-derived positional interval preserved PsO-like vs. PsA-like ordering in an independent transcriptomic cohort. Subject-level and subject–cell type observations were then classified as below-band (PsO-like), in-band (central reference zone), or above-band (PsA-like). Treatment sensitivity applications of the all-available score model are described in Section 4.11 and Supplementary Table S4.

PSX-enriched above-band compartments were defined as cell types in which PSX subjects were enriched in the above-band state relative to PsO subjects. For each cell type, this was tested only when at least six PsO and six PSX subjects with retained subject–cell type scores were available, using one-sided Fisher’s exact tests for enrichment of PSX in the above-band state relative to PsO and permutation testing based on 5000 shuffles of the PSX/PsO labels, followed by Benjamini–Hochberg correction across cell types.

### 4.8. Cell-Type Pathway State Modeling Along the CRS Continuum

To characterize within-cell-type pathway state remodeling associated with CRS, a focused Hallmark-plus-cytotoxicity pathway panel was evaluated in GSE194315 pseudobulk data. The panel included interferon-alpha response, interferon-gamma response, TNF/NFκB signaling, IL6/JAK/STAT3 signaling, inflammatory response, complement, IL2/STAT5 signaling, and TGF-β signaling, together with the published 8-gene cytotoxicity-associated transcript signature described by Turiello et al. (GZMA, GZMB, GZMH, GZMK, GZMM, GNLY, PRF1, and NKG7), which captures granzyme-, granulysin-, perforin-, and NKG7-associated cytotoxic effector programs [38]. The perforin/granzyme component is supported by the effector biology review by Voskoboinik et al. [39]. This signature was added because the Hallmark collection does not provide a dedicated cytotoxic lymphocyte effector pathway. Because the GSE194315 pseudobulk matrices used Ensembl gene identifiers, gene identifiers were mapped to gene symbols using the org.Hs.eg.db annotation package before ssGSEA scoring; duplicate mapped symbols were collapsed by retaining the highest-variance row.

The primary pathway analysis was restricted to a focused eight-cell-type panel selected for focused modeling on sufficiently represented immune compartments: CD8.Naive, NK, CD14.Mono, Treg, B.naive, CD4.Naive, CD4.TCM, and CD16.Mono. This analysis was intended as a focused rather than exhaustive survey of all annotated cell types. For each selected cell type, pseudobulk counts were normalized using edgeR::calcNormFactors and transformed to log-CPM values using cpm(log = TRUE, prior.count = 1), and ssGSEA pathway scores were then computed per subject using GSVA (v2.4.4). Cell types with fewer than eight subjects in common with the subject-level CRS table were excluded from the primary pathway analysis. A broader exploratory survey across all eligible annotated pseudobulk cell types in the GSE194315 untreated analysis set, defined here as cell types with at least eight subjects in common with the subject-level CRS table, is reported in Supplementary Figure S7 and Table S6; this broader screen was not used to redefine the primary eight-cell-type panel.

Within each selected cell type and pathway, pathway activity was modeled across the cross-sectional CRS continuum spanning PsO, PSX, and PsA subjects as a smooth function of $CRS_{\mathrm{norm}}$ using generalized additive models implemented in the mgcv package (v1.9-4) [40] of the form

$$\mathrm{pathway}\;\mathrm{score}∼s(CRS_{\mathrm{norm}},k=5).$$

For representative trajectory panels in Figure 3c,d and Supplementary Figure S3b,c, HC observations were displayed as points for visual context only, whereas the black smooths were fitted using pooled PsO, PSX, and PsA observations. HC observations were not used for GAM fitting or derivative-based inflection summaries. For each pathway–cell type pair, the pathway inflection point was operationally defined as the $CRS_{\mathrm{norm}}$ value at which the absolute first derivative of the fitted smooth function was maximal. This procedure was used as a descriptive summary of pathway-specific positional patterns across the cross-sectional CRS continuum.

### 4.9. Skin Transcriptomic Analysis in GSE186063

GSE186063 skin RNA-seq data were analyzed from a preprocessed dataset containing an edgeR DGEList, a logCPM expression matrix, and parsed GEO series-matrix metadata. Metadata rows were aligned to the expression matrix primarily by sample identifier, with fallback matching by sample title and GSM when necessary. Disease group, tissue state, and detailed unit group labels were standardized to AS-H, PsO-N, PsO-L, PsA-N, and PsA-L, where AS-H denotes healthy-appearing ankylosing spondylitis comparator skin rather than skin from unaffected healthy volunteers. Reliable donor-level pairing identifiers were not available from the public GEO sample fields; therefore, although paired lesional/non-lesional models had been prespecified for use only if at least five reliable pairs could be established, the final analysis-ready dataset used unpaired designs for all tested contrasts.

Differential expression analysis was performed using the edgeR quasi-likelihood framework with robust dispersion estimation. The tested contrasts were PsA-L vs. AS-H, PsA-N vs. AS-H, PsA-L vs. PsA-N, PsA-L vs. PsO-L, PsA-N vs. PsO-N, PsO-L vs. AS-H, PsO-N vs. AS-H, and PsO-L vs. PsO-N. For all contrasts, the design was specified as

∼0+group+sex+age.

group denotes the harmonized analysis group factor (AS-H, PsO-N, PsO-L, PsA-N, or PsA-L). Differential expression results were exported in full, and FDR-significant gene counts were summarized across contrasts.

Pathway analysis was performed from the differential expression results using pre-ranked GSEA with fgsea (v1.36.2) [41]. Ranking was based on signed $-\log_{10}\left(P\right)$ statistics derived from the edgeR quasi-likelihood test, defined as $\mathrm{sign}\left(\log FC\right)\times [-\log_{10}\left(P\right)]$, where *P* denotes the edgeR quasi-likelihood test *p* value. Hallmark, GO biological process, KEGG legacy, and Reactome collections were analyzed by GSEA. In addition, Hallmark over-representation analyses for upregulated and downregulated gene sets were performed using clusterProfiler::enricher (v4.18.4) [42] when at least 10 FDR-significant mapped genes were available in the corresponding direction, with all mapped tested genes in the corresponding contrast used as the background universe. For the primary skin axis scores, DIR_skin_ was anchored to the PsO-L vs. AS-H contrast and CRS_skin_ to the PsA-L vs. PsO-L contrast. Scores were computed by ssGSEA as UP minus DOWN using the same GSVA-based ssGSEA settings described in Section 4.6. Core signatures from the prespecified strict and relaxed GMT signature sets defined in the core-signature pipeline were used when available, with fallback first to the relaxed GMT set and then to ranked-signature TOP-100 sets when core signatures were unavailable or too small. This ranked-signature fallback was required for the primary CRS_skin_ score because the corresponding strict and relaxed core signatures were empty.

### 4.10. Exploratory Purified CD4^+^ T-Cell Methylation Analysis in GSE236694

GSE236694 was analyzed as an exploratory purified cell methylation cohort. Upstream preprocessing was performed from raw IDAT files using the same minfi Noob-based workflow described above, and the present analysis used the resulting analysis-ready beta-value, M value, and detection-*p* matrices. Differential methylation analysis was performed on M values using limma with robust empirical Bayes moderation and empirical Bayes trend modeling [30]. The design matrix included disease group and, when available and non-degenerate within the analyzed subset, age, sex, batch_slide, and batch_array. Three contrasts were evaluated: PsO vs. HC (DIR), PsA vs. PsO (CRS), and PsA vs. HC (disease spectrum).

DMRs were identified using DMRcate, with array type inferred automatically from probe identifiers. Primary DMR calling used CpG-level annotation with a strict seed threshold of FDR <0.05 and region calling under the default strict DMRcate setting (without relaxed pcutoff override). When no reportable regions were returned, pcutoff was relaxed sequentially to 0.05, 0.10, and then 0.20, while other DMRcate settings were held constant. In the reported run, all three contrasts yielded reportable regions at the first pcutoff relaxation step (pcutoff = 0.05), and no further relaxation was required [31]. Gene set enrichment was performed with missMethyl::gometh (v1.44.0) for GO and KEGG categories [43]. Because no contrast yielded FDR-significant CpG sets of sufficient size for enrichment, the reported gometh analyses used the fallback set of probes with nominal p<0.05.

Exploratory DIR and CRS methylation scores were constructed separately from the GSE236694 DMP tables for the DIR (PsO vs. HC) and CRS (PsA vs. PsO) contrasts using a tiered feature selection strategy, because the prespecified strict thresholds yielded too few FDR-significant CpGs to support stable score construction in this small cohort. The selection tiers were applied in the following order: (i) FDR ≤0.10 and |Δβ|≥0.05; (ii) FDR ≤0.20 and |Δβ|≥0.02; (iii) FDR ≤0.30 and |Δβ|≥0.01; (iv) fallback to top |Δβ| probes irrespective of FDR; and (v) fallback to top |t| probes. The top 30 CpGs passing the best available tier were used for each axis. In the reported GSE236694 score construction, both DIR and CRS used the top-|Δβ| fallback tier irrespective of FDR. For each probe, beta values were standardized relative to the appropriate baseline group (HC for DIR and PsO for CRS), multiplied by the sign of Δβ (or the sign of the *t* statistic if needed), averaged across probes, and then re-standardized within the baseline group. Because these scores were built and evaluated within the same small cohort, the resulting DIR and CRS scores from GSE236694 were interpreted only as exploratory within-cohort findings.

### 4.11. Additional Robustness Analyses

Additional robustness analyses quantified sensitivity to treatment annotation and band cutoff choices using previously generated GSE194315 score and pathway outputs. The all-available score model, which included medication-annotated subjects during signature learning and scoring, and a downstream exclusion application of that model, in which medication-annotated subjects were removed only after scoring, were compared with the primary untreated-analysis-set workflow. These comparisons summarized subject-level band occupancy, cell-type-level enrichment, and pathway-direction concordance.

CRS reference-band sensitivity was also assessed by applying alternative probability cut-point pairs (0.25/0.75, 0.30/0.70, and 0.33/0.67) to the GSE194315 untreated analysis set and summarizing subject-level above-band occupancy and PSX-enriched above-band compartments.

To refine interpretation of the PSX-enriched above-band Fisher analyses when PsO above-band counts were zero, Haldane–Anscombe continuity-corrected odds ratios and bounded Newcombe–Wilson risk difference confidence intervals were also computed as descriptive sensitivity summaries; these quantities did not replace the primary Fisher/permutation inference.

For the main text pathway state analyses, evaluated across the 72 pathway–cell type pairs in the primary pathway localization panel, pathway direction concordance was compared between the untreated analysis set and all-available outputs. These robustness and effect size summaries are reported in Supplementary Tables S4 and S5.

### 4.12. External Skin Cohort Checks in GSE205748 and GSE202011

Two additional public skin datasets were analyzed to provide external context for the limited additional PsA-related separation observed in GSE186063.

GSE205748 is a small independent bulk skin RNA-seq cohort containing healthy control skin, uninvolved PsA skin, and PsA lesional skin (9 samples per group) [22]. Gene-level count data were normalized with edgeR::calcNormFactors and transformed to logCPM values using cpm(log = TRUE, prior.count = 2) [35]. LogCPM expression values were then scored by ssGSEA against a focused Hallmark panel comprising TNF/NFκB signaling, interferon-alpha response, interferon-gamma response, IL6/JAK/STAT3 signaling, inflammatory response, and TGF-β signaling, using the same GSVA-based ssGSEA settings applied elsewhere in the manuscript [36]. The tested contrasts were PsA lesional versus healthy control skin and PsA lesional versus uninvolved PsA skin, evaluated by exact rank-sum permutation testing with Benjamini–Hochberg correction. Because the cohort did not include psoriasis without arthritis, it was used only for pathway-level PsA skin corroboration rather than for direct PsO versus PsA testing.

GSE202011 is an external spatial transcriptomic skin cohort containing healthy control, PsO, and PsA samples, including both lesional and non-lesional skin states [24]. Because the aim of this analysis was sample-level external projection of the GSE186063-derived bulk-like skin signatures rather than inference on spatial microenvironments, raw spot-level matrices were collapsed to sample-level pseudobulk counts by summing transcript counts across spots within each Visium sample. These sample-level pseudobulk counts were normalized with edgeR::calcNormFactors and transformed to logCPM values using cpm(log = TRUE, prior.count = 2) [35]. The official GSE186063-derived DIR_skin_ and CRS_skin_ signatures were then projected to these pseudobulk matrices using the same ssGSEA UP-minus-DOWN scoring logic and GSVA-based settings described above [36]. External score contrasts were evaluated by exact rank-sum permutation testing with Benjamini–Hochberg correction for the lesional comparisons PsO-L versus healthy control skin, PsA-L versus healthy control skin, and PsA-L versus PsO-L.

### 4.13. Covariate-Adjusted Consistency Checks

To verify that the principal directional conclusions were not explained solely by age or sex imbalance, we performed harmonized score-level consistency checks across the four core datasets. For selected key disease axis findings emphasized in the main figures, the relevant DIR/CRS score was fitted in a pairwise linear model including disease group, age, and sex:

score∼group+age+sex.

These checks were performed on the already-generated dataset-specific DIR/CRS score tables rather than by repeating each full modality-specific pipeline, and they were interpreted as harmonized sensitivity summaries rather than as replacements for the primary analyses. The resulting summary is provided in Supplementary Table S3.

### 4.14. Statistical Analysis and Software

Correlations between DIR and CRS were assessed using Pearson and Spearman coefficients. Multiple testing correction was performed using the Benjamini–Hochberg method where relevant. All tests were two-sided unless a directional alternative was explicitly specified (e.g., in the PSX-enriched above-band enrichment analysis).

Figure 3b–d and Supplementary Figure S3b,c should be interpreted as descriptive summaries of pathway state localization and representative fitted trajectories rather than as per-tile or per-curve hypothesis tests.

All analyses were performed in R version 4.5.1, and principal software packages and version numbers are reported at first use in the relevant subsections. During preparation of this study and manuscript, OpenAI GPT-5.4 Pro was used to assist with code review and debugging, as well as English-language editing and wording refinement. All analytical decisions, code execution, result verification, and final interpretation were performed and verified directly by the authors.

## 5. Conclusions

This study supports a model in which psoriasis without arthritis and psoriatic arthritis share a systemic inflammatory foundation but are separated by an additional immune state axis. DIR captures the common inflammatory component of psoriatic disease, whereas CRS captures multicompartment systemic reorganization associated with PsA. In the blood-derived analyses, the psoriasis–PsA distinction was represented more clearly by systemic molecular and cellular states than by skin inflammatory burden alone. These findings do not define an individual prognostic biomarker or a deployable diagnostic classifier, but they identify a testable systemic coordinate framework for future longitudinal and clinically annotated studies. Together, the results support the interpretation that PsA is distinguished from psoriasis by an additional, partly distinct systemic immune state configuration that is not adequately captured by cutaneous inflammatory burden alone.

## Data Availability

The public datasets analyzed in this study are available from the Gene Expression Omnibus (GEO) under accession numbers GSE200376, GSE194315, GSE186063, GSE236694, GSE205748, and GSE202011. Derived analysis tables supporting the findings of this study are provided within the article and its Supplementary Materials. Additional processed outputs and analysis scripts are available from the corresponding author upon reasonable request.

## Supplementary Materials

The following supporting information can be downloaded at: https://www.mdpi.com/article/10.3390/ijms27115121/s1.

## Abbreviations

The following abbreviations are used in this manuscript:

AUC	Area under the receiver operating characteristic curve
BMI	Body mass index
BSA	Body surface area
CASPAR	Classification Criteria for Psoriatic Arthritis
CRS	Contrast-resolved systemic state coordinate
DIR	Disease inflammatory response axis
DMARD	Disease-modifying antirheumatic drug
DMP	Differentially methylated probe
DMR	Differentially methylated region
FDR	False discovery rate
GAM	Generalized additive model
GEO	Gene Expression Omnibus
GO	Gene Ontology
GSEA	Gene set enrichment analysis
AS-H	Healthy-appearing ankylosing spondylitis comparator skin
HC	Healthy control
IDAT	Illumina intensity data file
IFN	Interferon
IRB	Institutional Review Board
KEGG	Kyoto Encyclopedia of Genes and Genomes
NK	Natural killer
PASI	Psoriasis Area and Severity Index
PBMC	Peripheral blood mononuclear cells
PsA	Psoriatic arthritis
PsO	Psoriasis without arthritis
PSX	Psoriasis with joint pain but without confirmed PsA (source-dataset label)
RNA-seq	RNA sequencing
ROC	Receiver operating characteristic
ssGSEA	Single-sample gene set enrichment analysis
TNF	Tumor necrosis factor
Treg	Regulatory T cell
UVB	Ultraviolet B

## Appendix A. Cross-Modality Interpretation of DIR and CRS

DIR and CRS were designed as harmonized biological coordinates rather than as identical assay-level variables across all datasets. Accordingly, the mathematical implementation of each score depended on the modality and cohort context, but the biological interpretation was held constant. The intended commonality was contrast-level: each modality independently instantiated DIR from the HC versus PsO comparison and CRS from the PsO versus PsA comparison using assay-appropriate features and scoring rules. Cross-modality transfer therefore relied on shared biological anchoring and PsO-relative positioning rather than on direct feature reuse or absolute scale equivalence. DIR was intended to capture the shared inflammatory disease component of the psoriatic spectrum, whereas CRS was intended to capture the additional systemic state associated with psoriasis versus PsA positioning.

In GSE200376, DIR and CRS were derived directly from weighted whole-blood methylation contrasts. DIR was anchored to the HC versus PsO comparison, and CRS was anchored to the PsA versus PsO comparison. In GSE194315, the same biological contrasts were implemented using subject-level pseudobulk transcriptomic signatures and ssGSEA-based up-minus-down scoring in the untreated analysis set. The GSE200376-derived CRS reference band was then applied to GSE194315 after cohort-specific normalization to the untreated PsO distribution, providing a shared positional frame for comparing subject and cell-type states along a PsO-like to PsA-like continuum.

In GSE186063, the same two-axis framework was adapted to the skin context. DIR_skin_ was anchored to the PsO-L versus AS-H contrast to represent inflammatory tissue burden, whereas CRS_skin_ was anchored to the PsA-L versus PsO-L contrast to test whether lesional skin within the same tissue state could distinguish arthritis-related biology beyond shared lesion activity. In GSE236694, DIR and CRS were constructed as exploratory purified cell methylation scores using the same biological contrast logic, but those analyses were interpreted cautiously because the same small cohort was used for both score construction and evaluation.

**Table A1:** Cross -modality implementation and interpretation of DIR and CRS.

Dataset	Biological Layer	DIR Implementation and Interpretation	CRS Implementation and Interpretation
GSE200376	Whole-blood DNA methylation	Weighted methylation score anchored to the HC versus PsO contrast; represents the shared inflammatory disease component across the psoriatic spectrum.	Weighted methylation score anchored to the PsA versus PsO contrast; represents the additional systemic state associated with PsA relative to PsO.
GSE194315	Subject-level PBMC single-cell RNA-seq	In the untreated analysis set, weighted average of cell-type ssGSEA up-minus-down transcriptomic scores learned from HC and PsO subjects; independently instantiates the same HC versus PsO contrast in the transcriptomic layer and positions systemic disease activity at the subject level.	In the same untreated analysis set, weighted average of cell-type ssGSEA up-minus-down transcriptomic scores learned from PsO and PsA subjects; independently instantiates the same PsO versus PsA contrast in the transcriptomic layer. After PsO-centered normalization, the GSE200376-derived band was used only as a positional reference to describe heterogeneous PSX subject-level positioning, identify PSX-enriched above-band compartments, and summarize pathway state remodeling along the CRS continuum.
GSE186063	Skin RNA-seq	ssGSEA up-minus-down score anchored to the PsO-L versus AS-H contrast, where AS-H denotes healthy-appearing ankylosing spondylitis comparator skin; represents cutaneous inflammatory burden.	ssGSEA up-minus-down score anchored to the PsA-L versus PsO-L contrast; tests whether lesional skin within the same tissue state carries additional PsA-related separation beyond shared lesion biology.
GSE236694	Purified CD4^+^ T-cell DNA methylation	Exploratory purified cell methylation score anchored to the PsO versus HC contrast; interpreted only as an exploratory within-cohort result because of the small cohort and limited CpG-level DMP support.	Exploratory purified cell methylation score anchored to the PsA versus PsO contrast; interpreted only as an exploratory within-cohort result and not as an independent validation layer.
