# Supplementary material for: A Systemic Immune State Axis Distinguishes Psoriatic Arthritis from Psoriasis
Source: Int J Mol Sci. 2026 Jun 5;27(11):5121. doi: 10.3390/ijms27115121 (PMC13257340; doi:10.3390/ijms27115121)
Supplement: Supplementary file 1 [file ijms-27-05121-s001.zip › supplementary_ijms_v8_tex_package/supplementary_ijms_v8_REFERENCE.pdf]

Supplementary Materials: A Systemic Immune-State Axis Distinguishes Psoriatic Arthritis from Psoriasis

Yoon Kyeong Lee [1,2], Hyun-A Seong[1,*]

1. Supplementary Figures

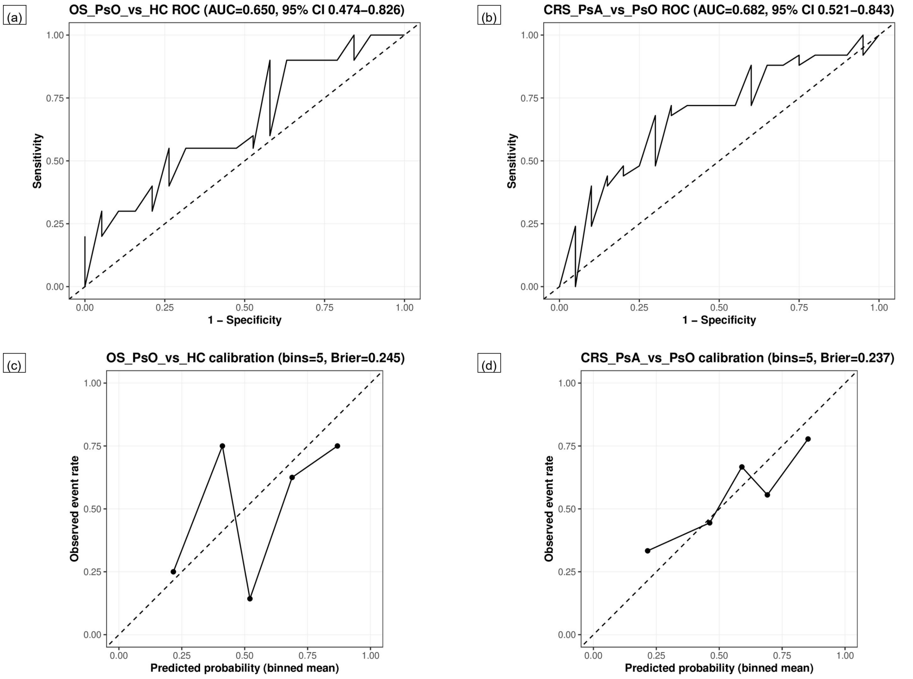

Figure S1. Internal leakage-aware stability and calibration assessment of the GSE200376 discovery framework. Panels (a) and (b) show receiver operating characteristic curves for DIR and CRS, respectively. Panels (c) and (d) show calibration plots for DIR and CRS, respectively. These results indicate modest discrimination and imperfect calibration, supporting interpretation of the discovery framework as a biological coordinate system rather than a clinical classifier. Relative to prevalence-only reference models, the corresponding Brier skill scores were 0.02 for DIR and 0.04 for CRS. Bootstrap summaries of the discovery-derived CRS reference band gave median lower and upper bounds of 1.50 and 2.10 (2.5th–97.5th percentiles 1.19–1.87 and 1.60–2.89), whereas repeated five-fold cross-validation gave median lower and upper bounds of 1.41 and 2.06 across 250 test folds.

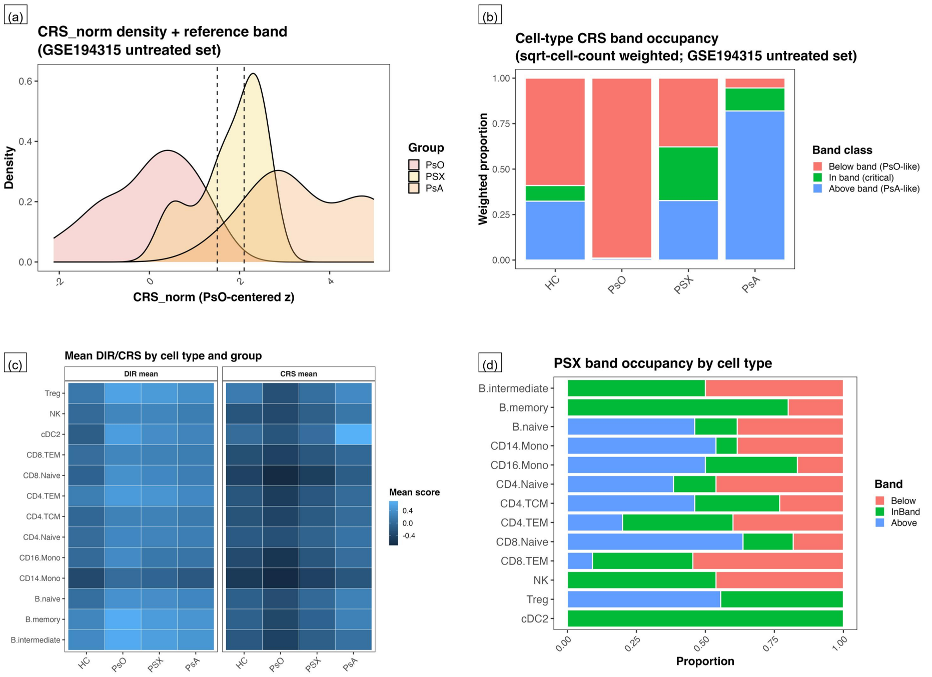

Figure S2. GSE194315 CRS-localization analyses in the untreated analysis set. (a) Density-based positioning of subjects along the discovery-derived CRS reference band. (b) Cell-type composition of below-band (PsO-like), in-band (central reference zone), and above-band (PsA-like) states. In panel (b), proportions were weighted by $\sqrt{n_{\text{cells}}}$ for each subject–cell type observation, balancing equal weighting with direct cell-count weighting. (c) Mean cell-type-level DIR and CRS scores across immune compartments. (d) PSX band occupancy by cell type. All-available sensitivity analyses, including the medication-annotated GSE194315 subjects excluded from the primary untreated analysis set, are summarized in Supplementary Table S4.

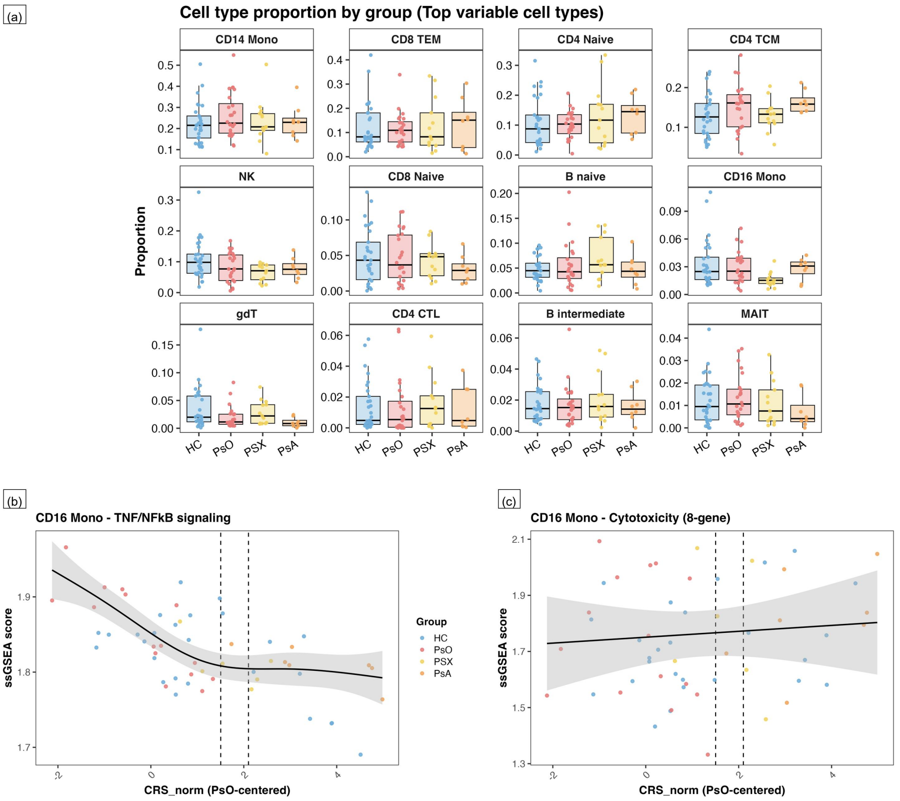

Figure S3. GSE194315 cell-type abundance and pathway-detail analyses in the untreated analysis set. (a) Top variable cell-type proportions across HC, PsO, PSX, and PsA. (b) CD16 monocyte TNF/NFκB signaling along the CRS continuum. (c) CD16 monocyte 8-gene cytotoxicity-signature activity along the CRS continuum. In panels (b,c), HC points are shown for visual context only, and black smooths show pooled PsO/PSX/PsA CRS-continuum GAM fits. These panels provide supplementary context for cell abundance and representative CD16 monocyte pathway-state patterns.

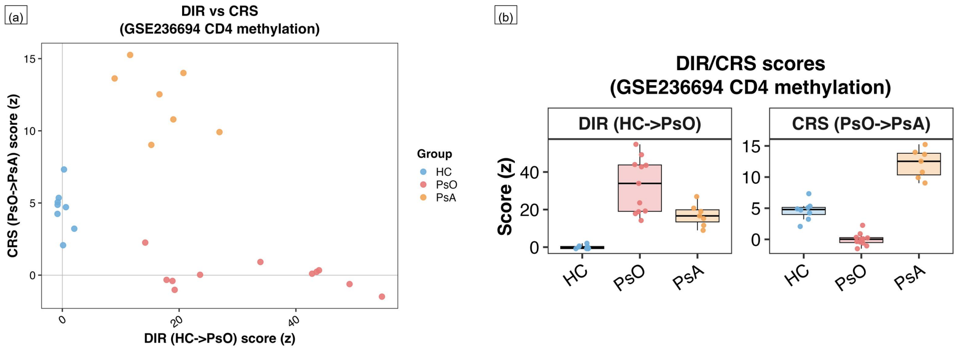

Figure S4. Exploratory purified CD4$^+$ T-cell methylation analysis in GSE236694. (a) DIR–CRS map across HC, PsO, and PsA samples. (b) Group-wise DIR and CRS distributions. These analyses provide only a small exploratory within-cohort view of the broader disease-axis framework.

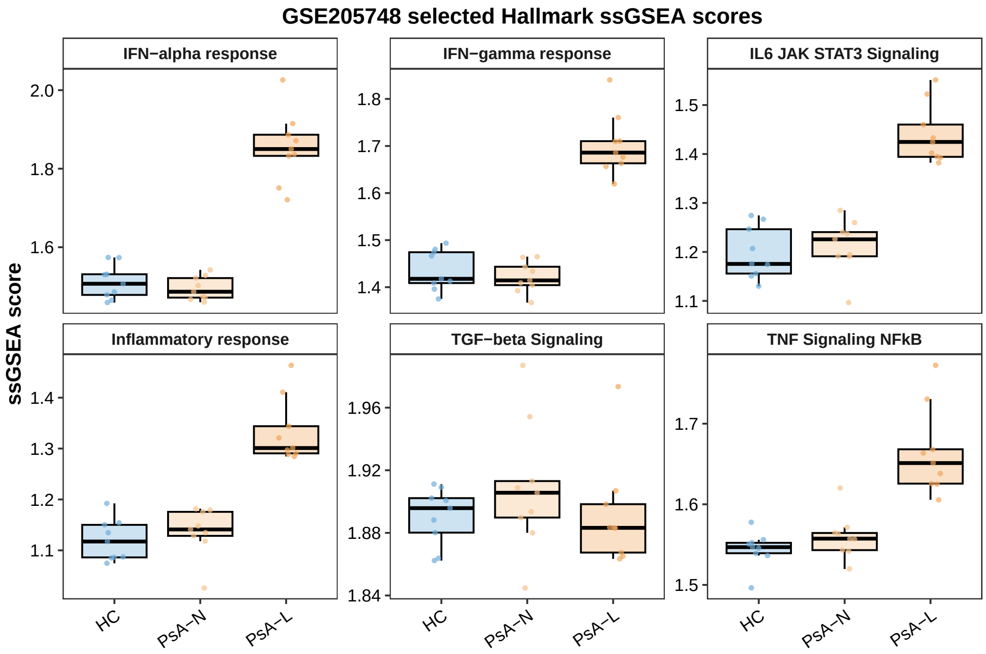

Figure S5. External PsA skin pathway corroboration in GSE205748. Selected Hallmark ssGSEA scores in a small independent bulk PsA skin RNA-seq cohort with 9 HC, 9 uninvolved PsA, and 9 lesional PsA samples show reproducible lesional enrichment of IL6/JAK/STAT3 signaling, inflammatory response, interferon-alpha response, interferon-gamma response, and TNF/NFκB signaling relative to both HC skin and uninvolved PsA skin by exact permutation rank-sum testing (all FDR $\leq 9.87 \times 10^{-5}$), whereas TGF-β signaling remained weak. This analysis was used only as pathway-level corroboration because the cohort did not include psoriasis without arthritis.

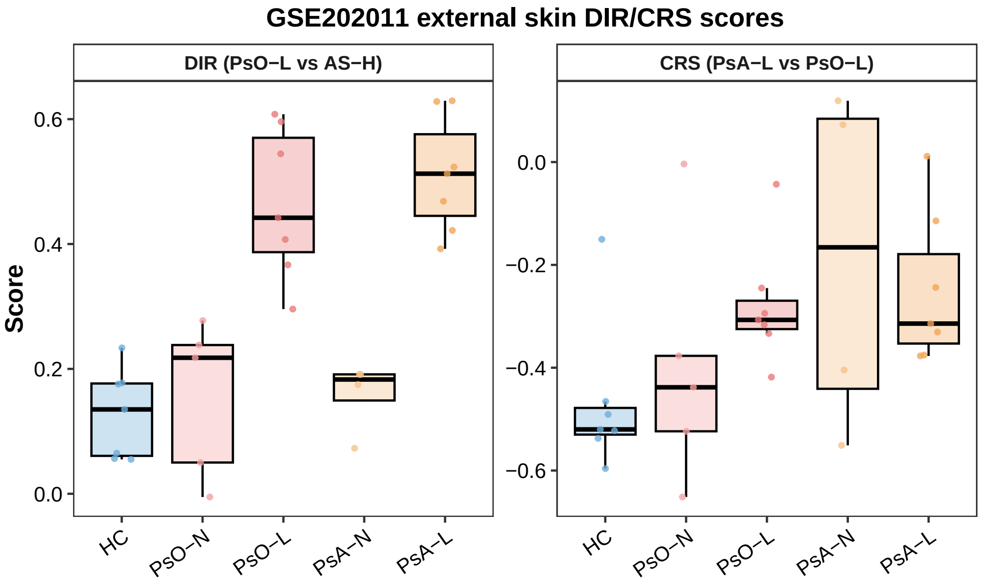

Figure S6. External spatial skin score check in GSE202011. GSE186063-derived DIR_{skin} and CRS_{skin} signatures were projected to sample-level spatial pseudobulk profiles from an independent psoriasis/PsA skin cohort because the aim was sample-level external score projection rather than inference on spatial microenvironments. Projected DIR_{skin} scores separated lesional PsO and lesional PsA from HC skin (both FDR = 1.75×10^{-3}) without additional lesional PsA-versus-PsO separation (FDR = 0.547). Projected CRS_{skin} scores showed only modest lesion-versus-HC shifts (FDR = 2.62×10^{-2} for PsO-L versus HC; FDR = 2.21×10^{-2} for PsA-L versus HC) and no lesional PsA-versus-PsO separation (FDR = 0.902). These external score-level checks therefore reinforced lesional inflammatory burden more clearly than any additional skin-based PsA-specific separation and were not consistent with stable external reproduction of lesional CRS_{skin} separation.

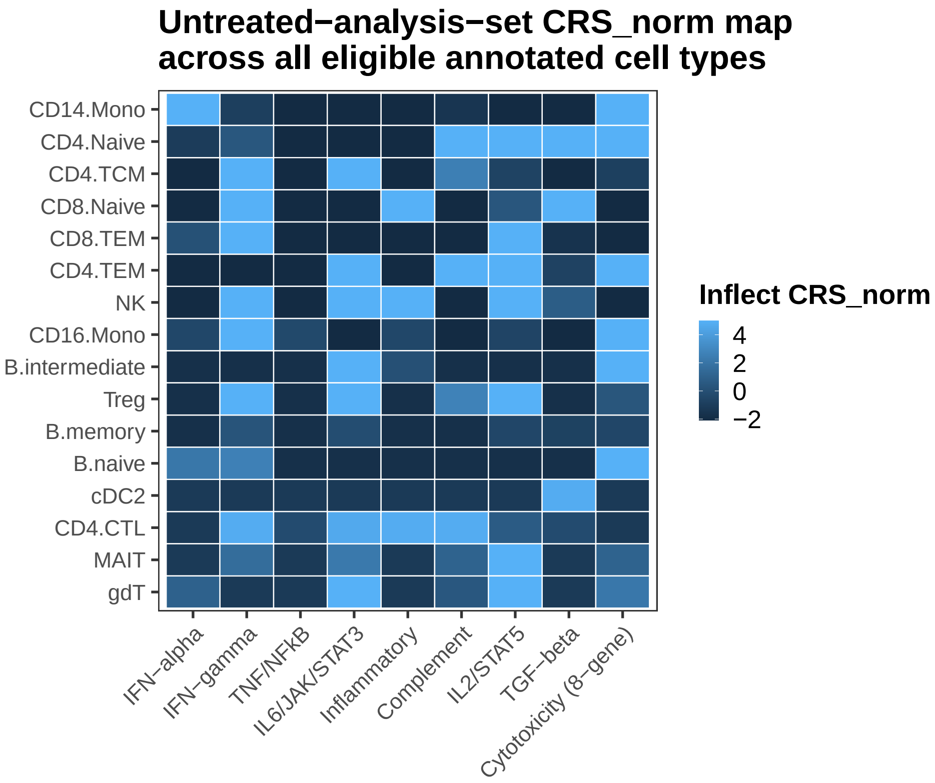

Figure S7. Exploratory all-cell-type pathway-state screen in the GSE194315 untreated analysis set. Using the same Hallmark programs, published 8-gene cytotoxicity signature, and CRS-normalized generalized additive modeling framework, we extended the pathway-localization screen to all eligible annotated pseudobulk cell types in the untreated analysis set. Among 21 annotated pseudobulk cell types, 17 met the minimum common-subject threshold for screening, and 16 yielded at least one estimable pathway maximal-change summary. This exploratory screen is retained as supplementary context only and was not used to redefine the main-text panel.

Table S1. Public datasets included in the cross-modality disease-axis analysis. The table summarizes the four core axis datasets and two external skin corroboration datasets, including cohort composition, demographic overview, and study role. For skin datasets with multiple biopsies or spatial sections per subject, group counts denote analysis-unit/sample counts, whereas age and sex summaries are reported at the subject level when available; therefore, totals may differ.

GEO Accession	Data Type	Source Material	Group / Phenotype	Age	Sex	Role in Current Study
GSE200376	Whole-blood DNA methylation	Peripheral whole blood	HC=19; PsO (psoriasis vulgaris)=20; PsA=25	48.6 ± 11.7 (range 29–68)	Male=48 Female=16	Primary discovery cohort used to define DIR and CRS.
GSE194315	Single-cell RNA sequencing with surface epitope profiling	Peripheral blood mononuclear cells	Untreated analysis set: HC=29; PsO (plaque psoriasis)=24; PSX (joint pain, no CASPAR PsA)=13; PsA (CASPAR)=8 All-available sensitivity: PSX=14; PsA=28	Untreated set: 45.4 ± 14.3 (range 22–79)	Untreated set: Male=34 Female=40	Independent systemic transcriptomic comparison cohort used for subject-level axis positioning, PSX positioning analysis, PSX-enriched above-band compartments, and pathway-state remodeling in the untreated analysis set; all-available outputs summarized in Supplementary Table S4 as treatment-sensitivity context.
GSE186063	Skin RNA sequencing	Lesional and non-lesional skin biopsies	AS-H=12; PsO-L/N (dermatologist-confirmed PsO)=13/13; PsA-L/N (CASPAR PsA)=13/15	44.0 ± 10.5 (range 20–63)	Male=25 Female=15	Cutaneous anchoring cohort used to assess whether shared inflammatory burden is better captured in skin than the psoriasis-versus-PsA distinction.
GSE236694	Purified $CD4^+$ T-cell DNA methylation	FACS-sorted $CD4^+$ T cells from PBMCs	HC=8; PsO (chronic plaque psoriasis)=11; PsA (Moll–Wright/CASPAR)=7	43.3 ± 18.5 (range 21–81)	Male=14 Female=12	Small exploratory purified-cell cohort used to examine whether disease-axis structure is also detectable in a more cell-intrinsic methylation context.
GSE205748	Skin RNA sequencing	Lesional and uninvolved PsA skin biopsies plus healthy control skin	HC=9; PsA-L/N (rheumatologist-diagnosed peripheral PsA)=9/9	NR	NR	Small external pathway-level corroboration cohort used to test whether inflammatory skin programs in PsA lesion samples were reproducible outside the primary skin dataset.
GSE202011	Spatial transcriptomics (Visium)	Lesional and non-lesional skin sections	HC=7; PsO-L/N (biologic-naive PsO)=7/5; PsA-L/N (biologic-naive PsA)=7/4	NR	NR	External score-projection cohort used to test whether GSE186063-derived DIR_{skin} and CRS_{skin} patterns generalized at the sample level in an independent spatial skin dataset.

Table S2. Clinical metadata availability, source-cohort notes, and ethics/consent information for the public datasets. NR indicates that the information was not reported in the harmonized public metadata records or in the reviewed source publications.

Dataset	Cohort Used	Source / Eligibility	Treatment Metadata	Activity / Duration	Ethnicity / Ancestry	Source-Reported Ethics / Notes
GSE200376	Whole-blood methylation cohort with HC, PsO, and PsA labels retained from GEO.	Second Xiangya Hospital of Central South University, Changsha, China. Detailed inclusion/exclusion criteria and PsA-domain definitions were NR.	GEO treatment protocol was annotated as "no" for all samples; medication class, washout, duration, dose intensity, and individual medication-history fields were NR.	Age and sex available. PASI, disease duration, age at onset, symptom duration, and PsA activity indices were NR.	NR.	The source article reported approval for peripheral blood collection by the Clinical Research Ethics Committee of the Second Xiangya Hospital, China, and written informed consent before sample collection.
GSE194315	PBMC single-cell cohort with HC, PsO, source-defined PSX, and CASPAR-classified PsA. PSX denotes psoriasis with joint complaints but no CASPAR PsA.	UCSF-associated clinics, San Francisco Bay Area, USA. Psoriasis was dermatologist-confirmed; PsA was rheumatologist-assessed and CASPAR-classified.	Drug-name-level systemic medication metadata were available. The primary analysis excluded one medication-annotated PSX subject and 20 medication-annotated PsA subjects. The all-available dataset was summarized in Supplementary Table S4 as treatment-sensitivity context and was not used for the primary GSE194315 results. Dose, duration, washout, treatment timing, and treatment response were NR.	Age, sex, BMI, joint-pain status, and PASI/BSA available for subsets. Disease duration, age at onset, symptom duration, and detailed PsA activity indices were NR.	White, Asian, Hispanic, African American, Other, and unreported categories available.	The source article reported UCSF IRB approval and written informed consent. Alternative causes of PSX joint pain and harmonized PsA domain-level annotations were NR.
GSE186063	Skin RNA-seq cohort with lesional and non-lesional PsO/PsA biopsies plus healthy-appearing AS comparator skin.	University Medical Center Utrecht, Netherlands. Original eligibility criteria were inherited from the source study and not re-adjudicated.	Sample-level treatment annotations were NR in GEO/harmonized records. The source article reported group-level treatment history, including DMARD, UVB, and biologic use within the 3 months before biopsy, but these variables were not available as sample-level covariates and were not modeled.	Age and sex available. PASI, disease duration, age at onset, symptom duration, and PsA activity indices were NR in analysis-ready metadata.	NR.	The source article reported Helsinki compliance, IRB approval before recruitment, ethical approval MvdL/nb/15/041945, and written informed consent. Skin-state contrasts were interpreted by biopsy state.
GSE236694	Purified $CD4^+$ T-cell methylation cohort with HC, chronic plaque psoriasis, and PsA labels retained from GEO.	Faculty of Medicine Carl Gustav Carus, TU Dresden, Germany. PsA was diagnosed using Moll–Wright and CASPAR criteria.	The source article reported no relevant systemic immunomodulating therapy at inclusion after prespecified washout; GEO treatment protocol was annotated as "no treatment" for the main analysis samples. Medication duration and dose intensity were NR.	Age, sex, and PASI available for patient samples. Disease duration, age at onset, symptom duration, and detailed PsA activity indices were NR.	Source article reported all patients as White European; sample-level ancestry was not available or modeled.	The source article reported ethics approval by the Faculty of Medicine Carl Gustav Carus, TU Dresden, and written informed consent. Small exploratory purified-cell cohort.
GSE205748	External skin RNA-seq check with healthy control skin, uninvolved PsA skin, and lesional PsA skin.	Glasgow, UK. PsA participants had rheumatologist-diagnosed peripheral arthritis and active skin disease; only axial or enthesial disease was excluded.	The source article reported no biologic therapy or phototherapy; non-biologic systemic treatment was reported in a subset. Per-sample medication covariates were not modeled in this small pathway-level check.	Time since diagnosis, tender/swollen joint counts, and PASI were reported in the source article but not modeled as covariates here.	NR.	The source article reported West of Scotland Research Ethics Service approval and written informed consent. External PsA skin corroboration only; PsO was not represented.
GSE202011	External spatial transcriptomic skin check with HC, PsO, and PsA samples across lesional and non-lesional skin states.	NYU Langone Health, New York, USA. Skin-biopsy cohort from healthy controls and biologic-naive psoriatic-disease patients.	GEO/source records indicate biologic-naive psoriatic-disease samples. Non-biologic medication, treatment duration, and dose intensity were NR.	Cutaneous severity stratification was used in the source study; harmonized duration fields and PsA activity indices were NR for the current projection.	NR.	The source article/GEO records reported NYU Langone IRB-approved protocols S20-01167 and S12-00831 for skin biopsies; explicit consent wording was not present in the reviewed article text. Sample-level projection only.

Table S3. Harmonized age- and sex-adjusted summary of the key disease-axis findings across the four core datasets.

Dataset	Biological Layer	Key Finding Tested	Axis	Adjusted Model	Beta	SE	P Value	Result Summary
GSE200376	Whole-blood DNA methylation	DIR captures the shared inflammatory-response axis of psoriasis	DIR_z (PsO vs HC)	$DIR_z \sim$ group + Age + Sex	1.336	0.246	4.27E-06	Retained after age/sex adjustment
GSE200376	Whole-blood DNA methylation	CRS distinguishes PsA from psoriasis in the systemic discovery cohort	CRS_z (PsA vs PsO)	$CRS_z \sim$ group + Age + Sex	1.696	0.154	7.36E-14	Retained after age/sex adjustment
GSE194315	Subject-level PBMC single-cell RNA-seq	DIR tracks shared psoriatic disease activity in untreated GSE194315	DIR_overall (PsO vs HC)	DIR_overall $\sim$ group + Age + Sex	0.311	0.034	3.86E-12	Retained after age/sex adjustment
GSE194315	Subject-level PBMC single-cell RNA-seq	CRS positions PSX between PsO and PsA on average in untreated GSE194315	CRS_overall (PSX vs PsO)	CRS_overall $\sim$ group + Age + Sex	0.262	0.048	4.37E-06	Retained after age/sex adjustment, but subject-level above-band occupancy was attenuated
GSE194315	Subject-level PBMC single-cell RNA-seq	CRS is higher in untreated PsA than in psoriasis	CRS_overall (PsA vs PsO)	CRS_overall $\sim$ group + Age + Sex	0.505	0.065	1.81E-08	Retained after age/sex adjustment; internal anchoring only
GSE186063	Skin RNA-seq	Skin transcriptomes strongly capture lesional inflammatory burden	DIR_skin (PsO-L vs AS-H)	DIR_skin $\sim$ group + Age + Sex	0.524	0.058	1.00E-08	Retained after age/sex adjustment
GSE186063	Skin RNA-seq	Lesional skin shows limited additional DIR separation between psoriasis and PsA within the same tissue state	DIR_skin (PsA-L vs PsO-L)	DIR_skin $\sim$ group + Age + Sex	0.034	0.068	0.6186	No significant additional DIR shift after age/sex adjustment
GSE186063	Skin RNA-seq	Lesional skin retains a modest but significant CRS shift between psoriasis and PsA within the same tissue state	CRS_skin (PsA-L vs PsO-L)	CRS_skin $\sim$ group + Age + Sex	0.284	0.078	0.001398	Modest but significant CRS shift retained after age/sex adjustment
GSE236694	Purified $CD4^+$ T-cell DNA methylation	Exploratory cell-intrinsic DIR consistency check	DIR_z (PsO vs HC)	$DIR_z \sim$ group + Age + Sex	37.323	5.575	7.18E-06	Exploratory direction retained after age/sex adjustment; same-cohort only
GSE236694	Purified $CD4^+$ T-cell DNA methylation	Exploratory cell-intrinsic CRS consistency check	CRS_z (PsA vs PsO)	$CRS_z \sim$ group + Age + Sex	11.745	0.795	6.19E-10	Exploratory direction retained after age/sex adjustment; same-cohort only

Note: For comparability, dataset-specific DIR/CRS score tables were analyzed with pairwise linear models including disease group, age, and sex. This table is intended as a consistency check showing that the principal directional findings were retained after age/sex adjustment and does not replace the primary modality-specific analyses reported in the main text. GSE236694 remains exploratory because the score was derived within the same small cohort, CpG-level DMP support was limited, and the resulting score-level checks remain within-cohort observations. Because the GSE236694 scores were constructed and re-standardized within that same small cohort, the resulting regression coefficients are not directly comparable in magnitude to coefficients from the other modalities. For GSE236694, DIR_z and CRS_z denote baseline-referenced exploratory scores after within-cohort score construction and should not be interpreted as standardized effect-size coefficients.

Table S4. Treatment-sensitivity and pathway-direction robustness analyses for GSE194315.

**Analysis**	**Variants Tested**	**Primary Summary**	**Interpretation**
Untreated-analysis-set subject-level occupancy	Subjects with recorded systemic medication excluded before GSE194315 signature learning, scoring, band assignment, and pathway modeling	The untreated analysis set contained 29 HC, 24 PsO, 13 PSX, and 8 PsA subjects. Above-band occupancy was PsO 0/24, PSX 7/13, and PsA 7/8; PSX subjects were heterogeneous, with 7/13 above-band, 3/13 in-band, and 3/13 below-band.	PsO-versus-PsA anchoring was preserved in the untreated analysis set, but the PSX subject-level above-band result was attenuated and should not be treated as a strong primary claim.
CRS reference-band probability-cutoff sensitivity	Alternative GSE200376-derived probability pairs of 0.25/0.75, 0.30/0.70, and 0.33/0.67 applied to the GSE194315 untreated analysis set	Across the main 0.20/0.80 band and alternative 0.25/0.75, 0.30/0.70, and 0.33/0.67 bands, untreated GSE194315 subject-level above-band occupancy was unchanged: PsO 0/24, PSX 7/13, and PsA 7/8. Seven FDR-significant PSX-enriched above-band compartments were retained across all cutoff pairs.	The subject-level band assignment and cell-type enrichment summaries were not driven by the specific heuristic 0.20/0.80 probability cut points; the band remains a positional reference interval rather than an optimized diagnostic threshold.
All-available and downstream-exclusion sensitivity	All-available score model; the same score model applied after excluding medication-annotated subjects downstream	The all-available sensitivity analysis yielded PsO/PSX/PsA above-band occupancy of 0/24, 8/14, and 21/28. Applying the same all-available score model only after excluding medication-annotated subjects yielded PsO/PSX/PsA above-band occupancy of 0/24, 8/13, and 6/8.	Simple downstream exclusion of treated subjects is not equivalent to defining the CRS signature from untreated PsO and untreated PsA subjects. The all-available result is therefore reported as treatment-sensitivity context.
Untreated-analysis-set cell-type enrichment	One-sided Fisher exact tests and 5000-label permutation tests for PSX above-band enrichment relative to PsO	FDR-significant PSX-enriched above-band compartments included CD8 naive T cells, CD4 central memory T cells, CD14 monocytes, B naive cells, CD4 naive T cells, Treg cells, and CD16 monocytes.	Cell-type-level enrichment remained detectable in the untreated analysis set even though the subject-level PSX above-band count was attenuated.
Pathway-direction sensitivity	Pathway summaries from the primary untreated workflow compared with all-available outputs	Across 72 pathway–cell type pairs, untreated-analysis-set versus all-available Spearman directions were concordant for 60 pairs (83.3%; median absolute ρ difference 0.083). GAM slope directions were concordant for 65 pairs (90.3%; median absolute inflection shift 0.708 normalized CRS units).	Pathway summaries were more stable at the level of directional localization than at the level of exact inflection-point placement.
Treatment-annotation limitation	Source metadata review	GSE194315 medication metadata provided drug names only. Dose, duration, washout, treatment timing, treatment response, and pretreatment disease severity were not available in the public records.	Defining the primary GSE194315 workflow within the untreated analysis set reduces direct treatment confounding but also reduces the untreated PsA learning set to eight subjects and cannot fully reconstruct pretreatment biology.

Table S5. Continuity-corrected effect-size summaries for PSX-enriched above-band compartments in the GSE194315 untreated analysis set.

Cell Type	PsO Above/Total	PSX Above/Total	Haldane–Anscombe OR (95% CI)	Risk Difference (95% CI)	Fisher FDR
CD8 naive T cells	0/18	7/11	61.7 (2.94–1292.30)	0.636 (0.304–0.848)	2.54×10^{-3}
CD4 central memory T cells	0/24	6/13	42.5 (2.13–844.87)	0.462 (0.194–0.709)	4.40×10^{-3}
CD14 monocytes	1/24	7/13	18.1 (2.56–127.88)	0.497 (0.202–0.729)	4.40×10^{-3}
B naive cells	0/20	6/13	35.5 (1.78–710.56)	0.462 (0.181–0.709)	4.65×10^{-3}
CD4 naive T cells	0/24	5/13	31.7 (1.58–635.78)	0.385 (0.135–0.645)	7.09×10^{-3}
Treg cells	0/10	5/9	25.7 (1.16–568.91)	0.556 (0.155–0.811)	2.17×10^{-2}
CD16 monocytes	0/15	3/6	31.0 (1.29–747.03)	0.500 (0.127–0.812)	2.58×10^{-2}

Note: Exact Fisher odds ratios were infinite for rows in which no PsO subjects occupied the above-band state. Haldane–Anscombe continuity correction and bounded Newcombe–Wilson risk-difference confidence intervals are reported here as descriptive effect-size summaries and do not replace the main Fisher/permutation inference.

Table S6. Exploratory all-cell-type pathway-screen eligibility and directional summaries in the GSE194315 untreated analysis set.

Cell Type	Common Subjects	Eligibility	Pathway Pairs	Median |Slope|	Up	Down
B intermediate cells	42	Included	9	0.0155	6	3
B memory cells	43	Included	9	0.0066	1	8
B naive cells	66	Included	9	0.0112	3	6
CD14 monocytes	74	Included	9	0.0281	1	8
CD16 monocytes	54	Included	9	0.0045	3	6
CD4 CTL cells	27	Included	9	0.0038	4	5
CD4 central memory T cells	74	Included	9	0.0053	2	7
CD4 effector memory T cells	56	Included	9	0.0057	4	5
CD4 naive T cells	72	Included	9	0.0041	4	5
CD8 effector memory T cells	70	Included	9	0.0038	2	7
CD8 naive T cells	59	Included	9	0.0069	4	5
Conventional dendritic cells 2	27	Included	9	0.0059	3	6
MAIT cells	28	Included	9	0.0061	1	8
NK cells	72	Included	9	0.0020	3	6
Treg cells	44	Included	9	0.0029	3	6
$\gamma\delta$ T cells	41	Included	9	0.0023	2	7
CD8 central memory T cells	13	Included	0	—	0	0
NK CD56^{bright} cells	6	Excluded	0	—	0	0
Plasmablasts	1	Excluded	0	—	0	0
Platelets	5	Excluded	0	—	0	0
pDCs	5	Excluded	0	—	0	0

Note: The exploratory all-cell-type screen used the same Hallmark plus published 8-gene cytotoxicity signature, CRS-normalized generalized additive modeling workflow, and minimum common-subject threshold ($n \geq 8$) as the focused pathway analysis, but it was generated from the GSE194315 untreated analysis set. Pathway pairs denote estimable maximal-change summaries; Up and Down indicate the direction of those summaries along the CRS continuum. Median |Slope| summarizes the median absolute fitted GAM slope across estimable pathway summaries for each cell type and is used only as a descriptive magnitude index. Of 21 annotated pseudobulk cell types, 17 were eligible for exploratory screening and 16 yielded at least one estimable pathway maximal-change summary. NK CD56^{bright} cells, plasmablasts, platelets, and pDCs were excluded because fewer than eight untreated subjects were matched to the subject-level CRS table; CD8 central memory T cells met the subject-count threshold but yielded no estimable pathway pair. The primary focused panel was predefined separately and was not redefined by this exploratory screen.
