# Supplementary material for: A Systemic Immune State Axis Distinguishes Psoriatic Arthritis from Psoriasis
Source: Int J Mol Sci. 2026 Jun 5;27(11):5121. doi: 10.3390/ijms27115121 (PMC13257340; doi:10.3390/ijms27115121)
Supplement: Supplementary file 1 [file ijms-27-05121-s001.zip › supplementary_ijms_v8_tex_package/Definitions/Supplementary_Figure_S3_GSE194315_Untreated_Additional_Details.pdf]

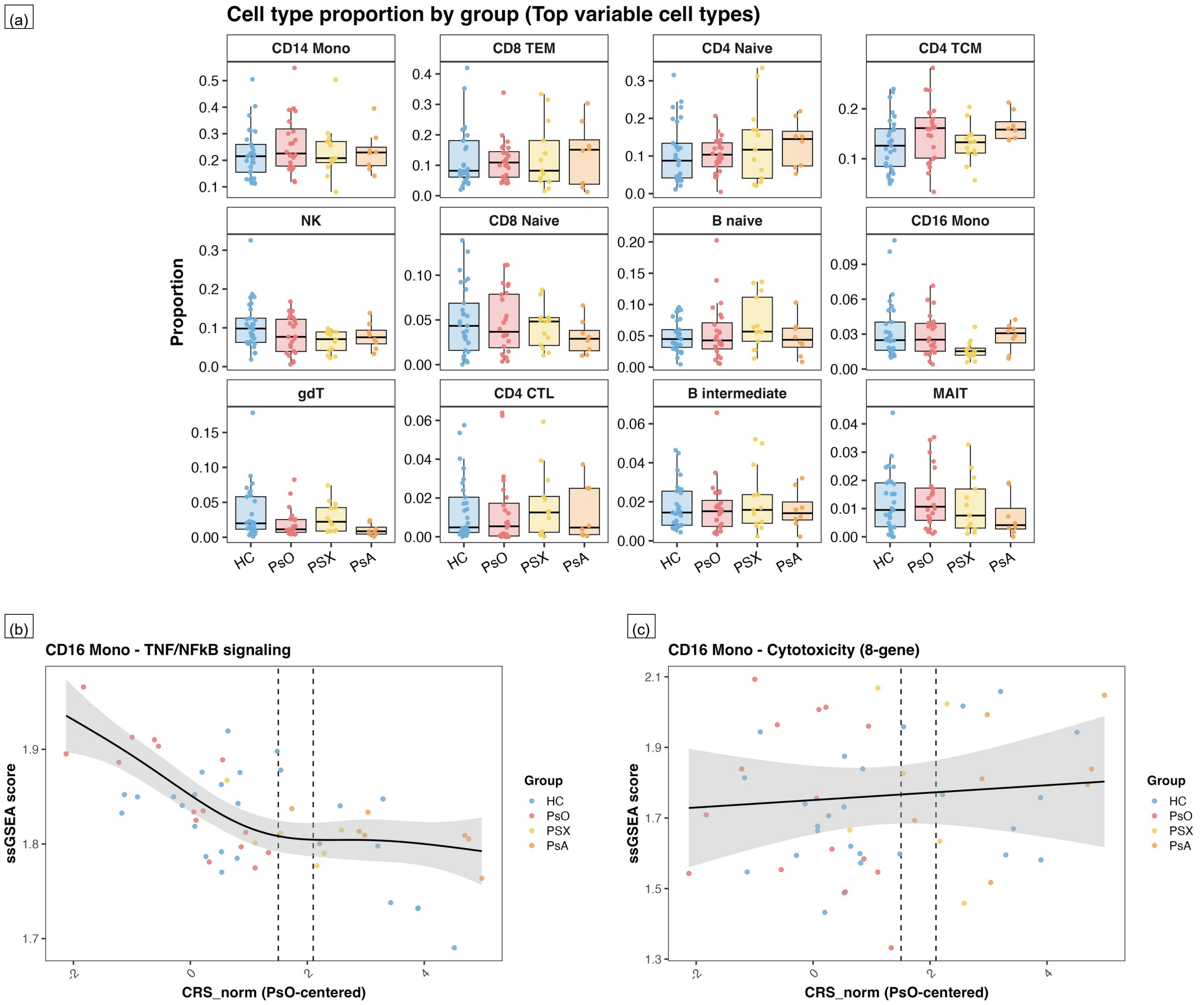

(a)
Cell type proportion by group (Top variable cell types)
CD14 Mono
CD8 TEM
CD4 Naive
CD4 TCM
NK
CD8 Naive
B naive
CD16 Mono
gdT
CD4 CTL
B intermediate
MAIT
Proportion
HC
PsO
PSX
PsA
(b)
CD16 Mono - TNF/NFkB signaling
ssGSEA score
CRS_norm (PsO-centered)
Group
HC
PsO
PSX
PsA
(c)
CD16 Mono - Cytotoxicity (8-gene)
ssGSEA score
CRS_norm (PsO-centered)
Group
HC
PsO
PSX
PsA
