# Supplementary material for: A Systemic Immune State Axis Distinguishes Psoriatic Arthritis from Psoriasis
Source: Int J Mol Sci. 2026 Jun 5;27(11):5121. doi: 10.3390/ijms27115121 (PMC13257340; doi:10.3390/ijms27115121)
Supplement: Supplementary file 1 [file ijms-27-05121-s001.zip › supplementary_ijms_v8_tex_package/Definitions/Supplementary_Figure_S2_GSE194315_Untreated_Transition_Details.pdf]

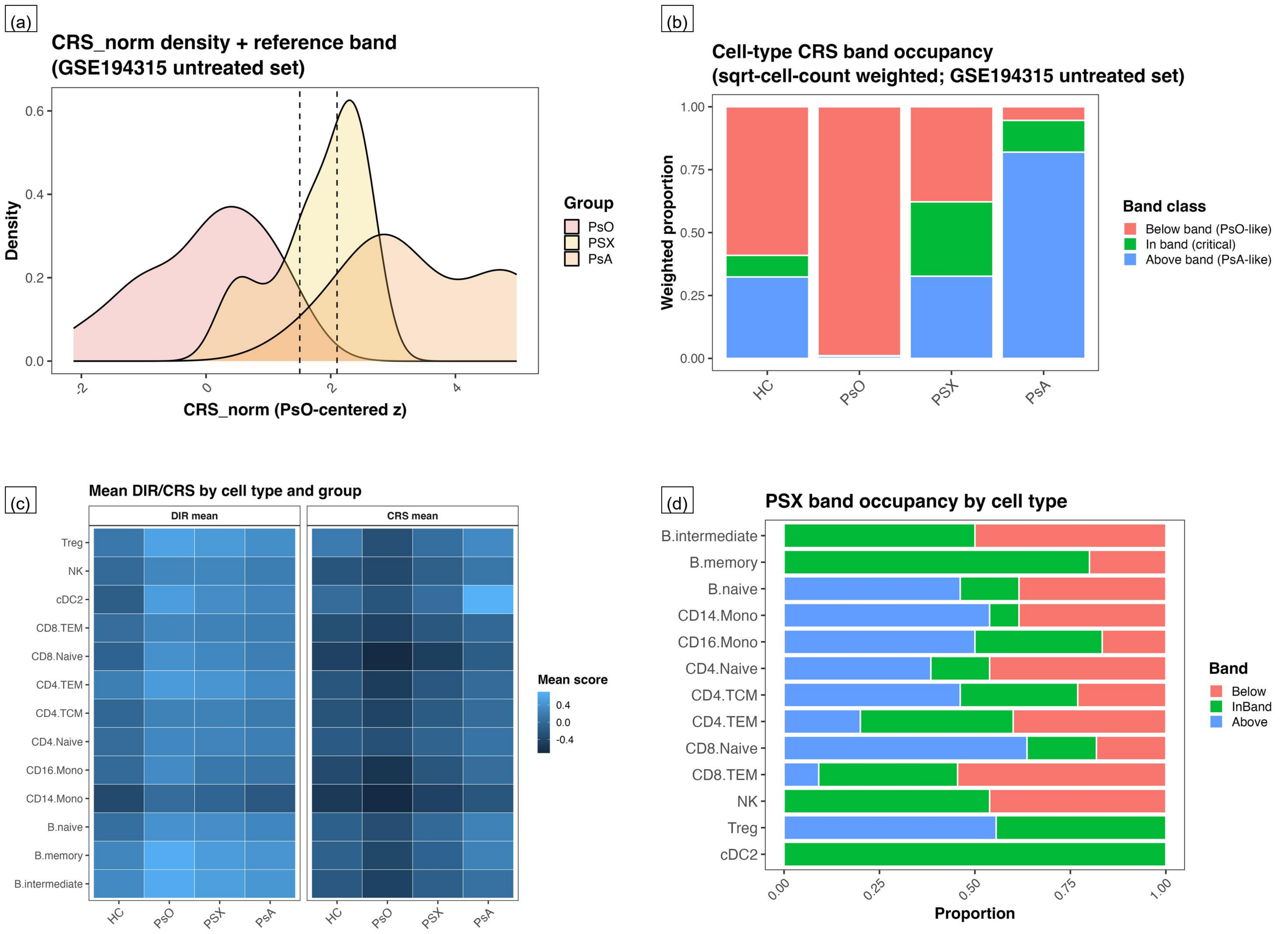
(a)
CRS_norm density + reference band
(GSE194315 untreated set)
Density
CRS_norm (PsO-centered z)
Group
PsO
PSX
PsA
(b)
Cell-type CRS band occupancy
(sqrt-cell-count weighted; GSE194315 untreated set)
Weighted proportion
Band class
Below band (PsO-like)
In band (critical)
Above band (PsA-like)
HC
PsO
PSX
PsA
(c)
Mean DIR/CRS by cell type and group
DIR mean
CRS mean
Mean score
Treg
NK
cDC2
CD8.TEM
CD8.Naive
CD4.TEM
CD4.TCM
CD4.Naive
CD16.Mono
CD14.Mono
B.naive
B.memory
B.intermediate
(d)
PSX band occupancy by cell type
Proportion
Band
Below
InBand
Above
