# Supplementary figures and images for: A Systemic Immune State Axis Distinguishes Psoriatic Arthritis from Psoriasis
Source: Int J Mol Sci. 2026 Jun 5;27(11):5121. doi: 10.3390/ijms27115121 (PMC13257340; doi:10.3390/ijms27115121)

# GSE205748 selected Hallmark ssGSEA scores

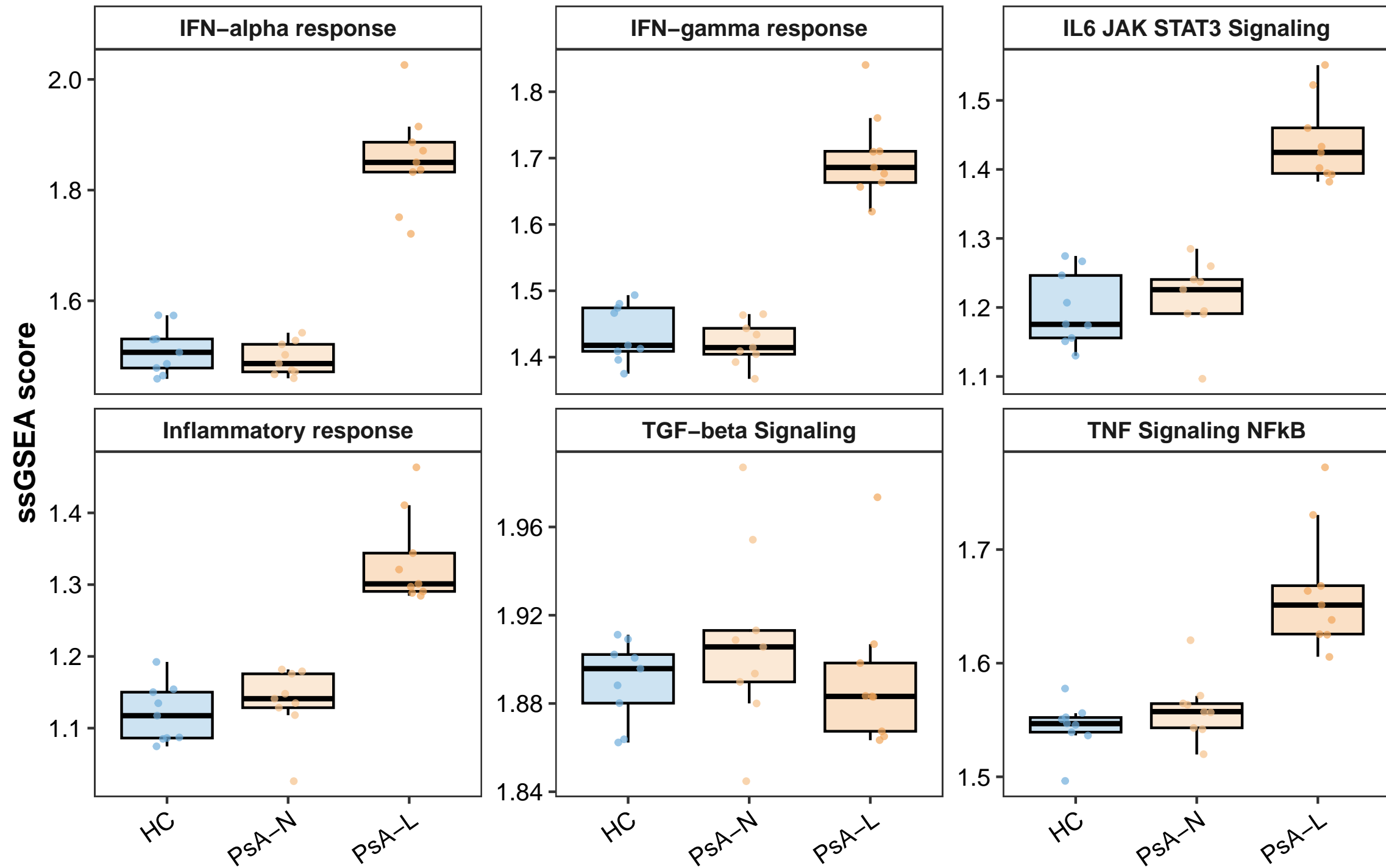

Supplement: Supplementary file 1 [file ijms-27-05121-s001.zip › supplementary_ijms_v8_tex_package/Definitions/Supplementary_Figure_S5_GSE205748_Skin_Corroboration_v3.pdf]

# GSE202011 external skin DIR/CRS scores

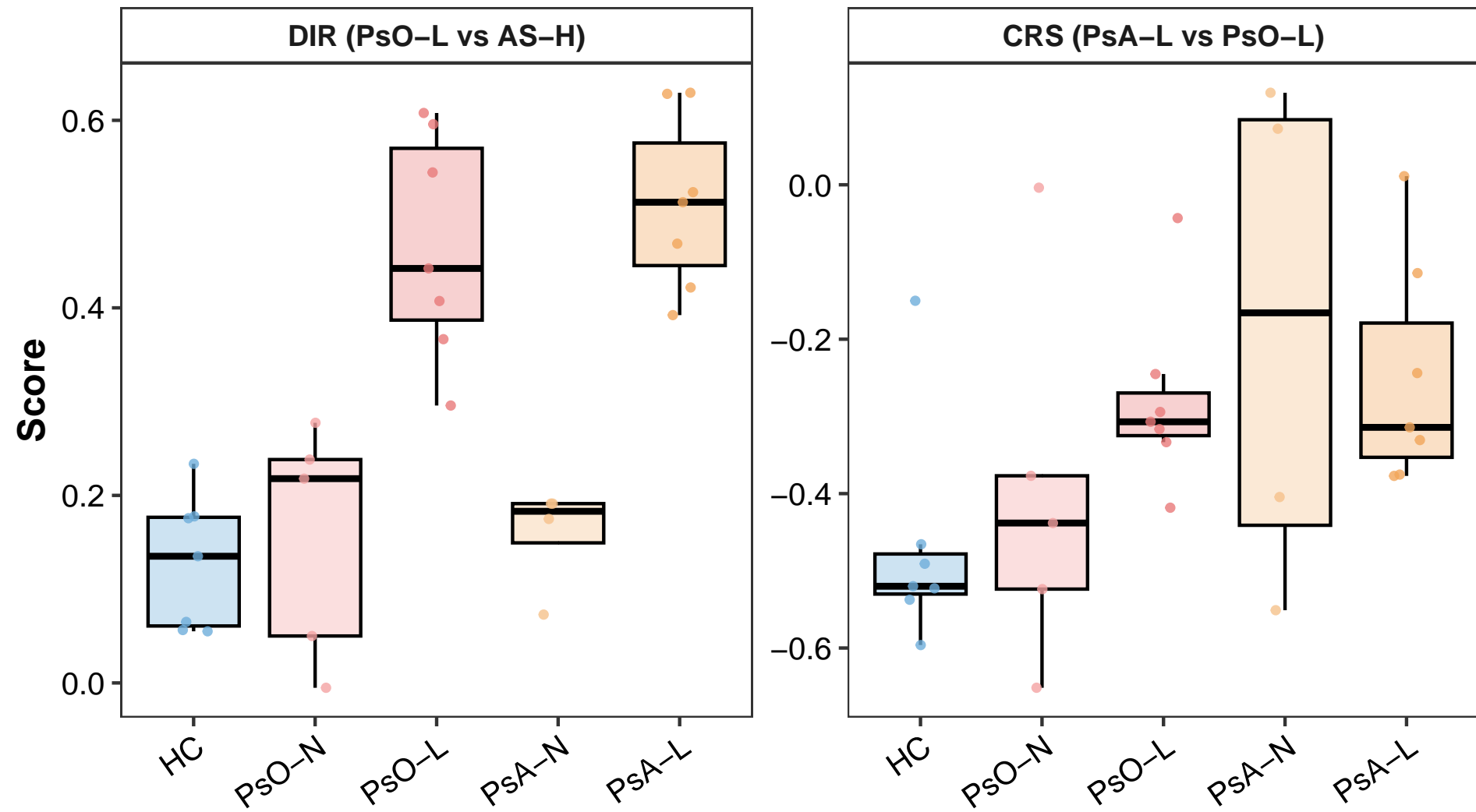

Supplement: Supplementary file 1 [file ijms-27-05121-s001.zip › supplementary_ijms_v8_tex_package/Definitions/Supplementary_Figure_S6_GSE202011_External_Skin_Check_v3.pdf]

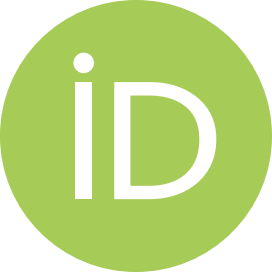

Supplement: Supplementary file 1 [file ijms-27-05121-s001.zip › supplementary_ijms_v8_tex_package/Definitions/logo-orcid.pdf]

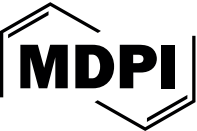

Supplement: Supplementary file 1 [file ijms-27-05121-s001.zip › supplementary_ijms_v8_tex_package/Definitions/logo-mdpi-eps-converted-to.pdf]

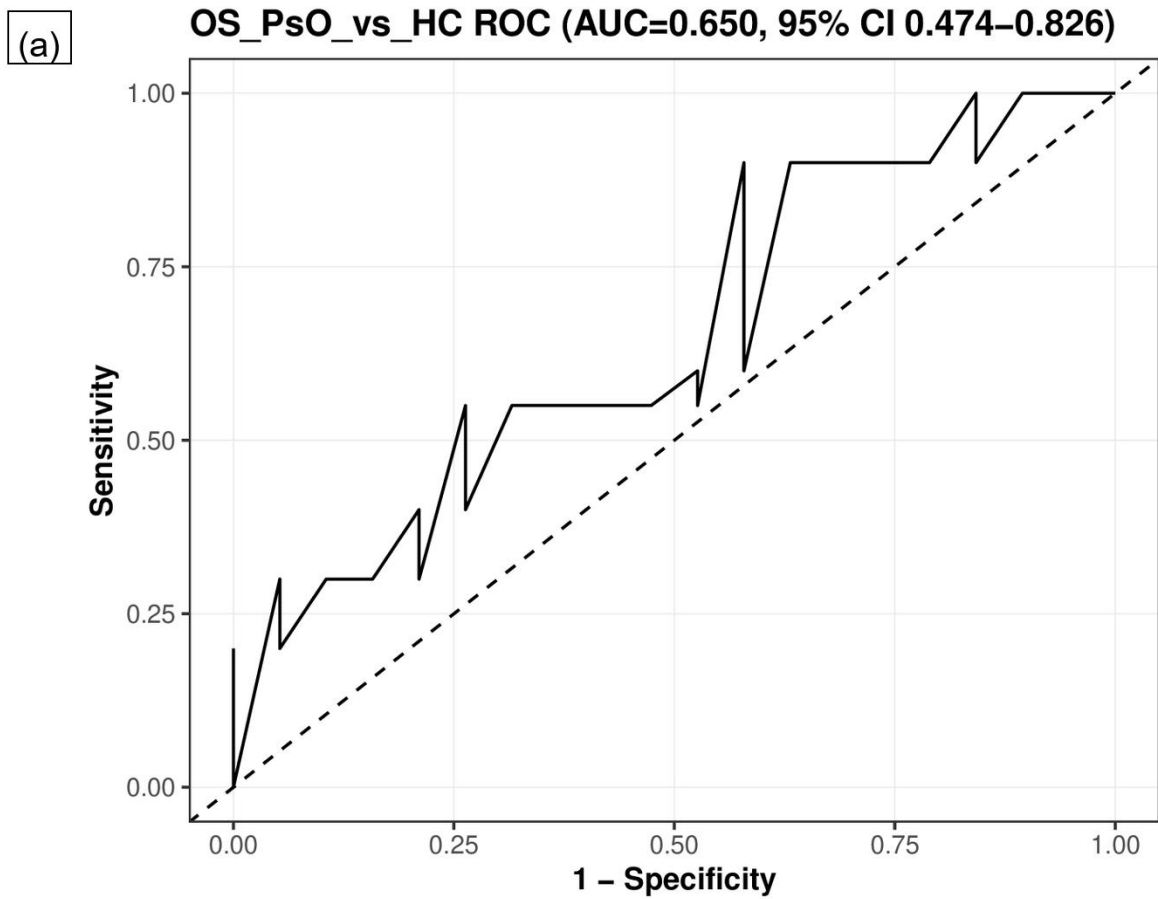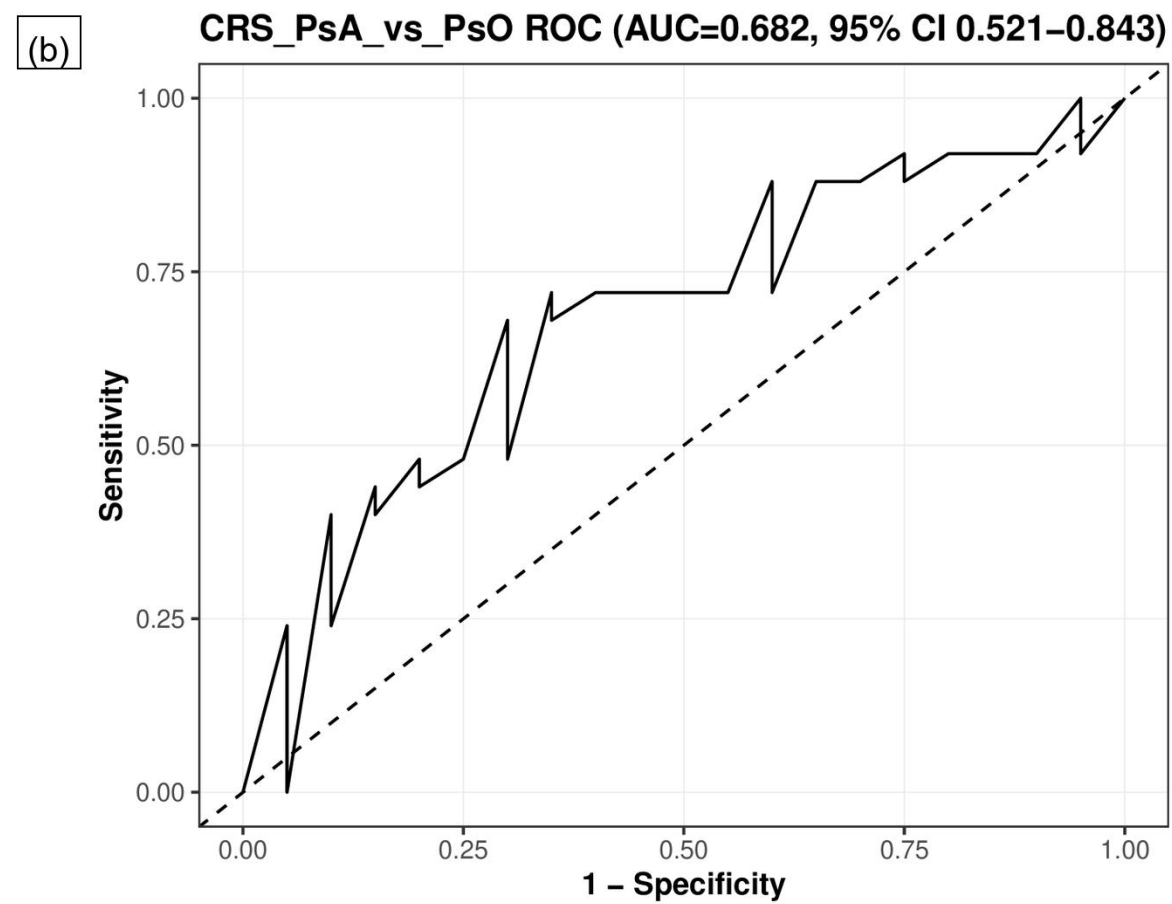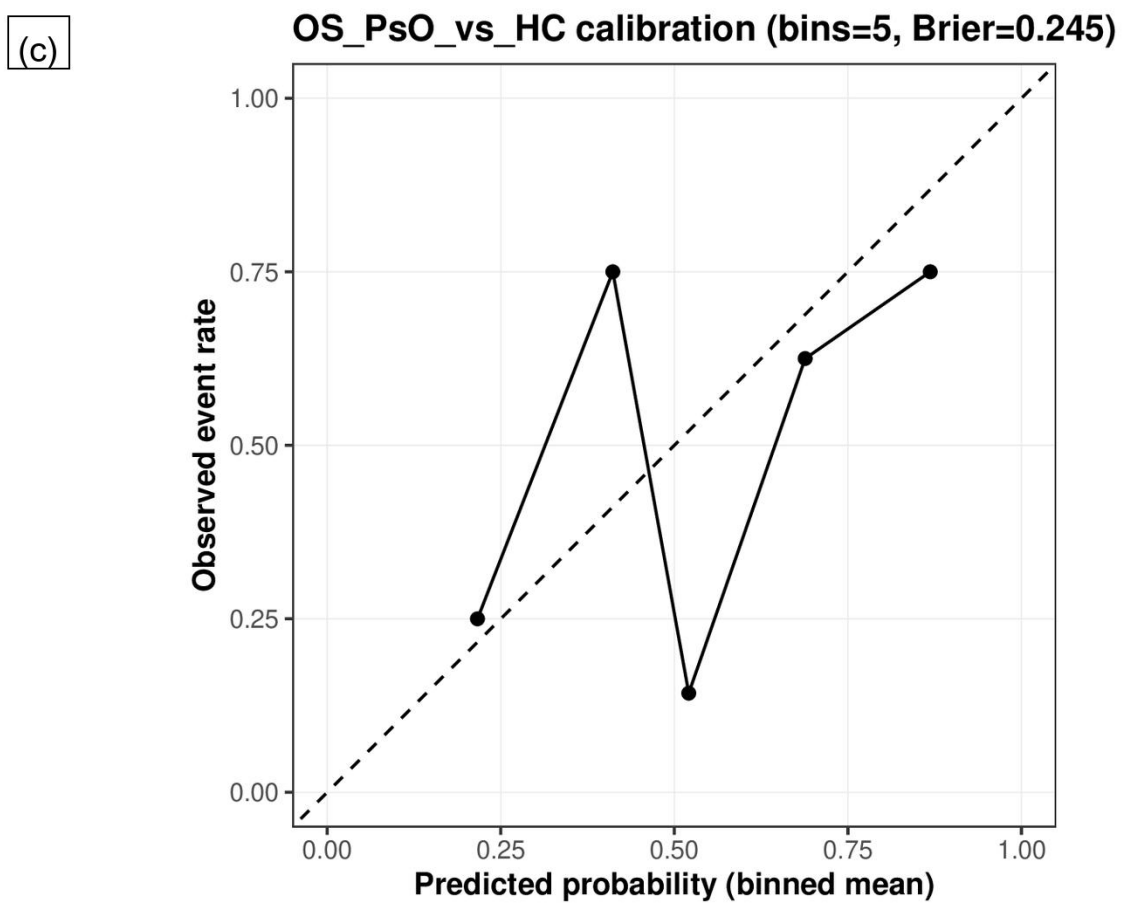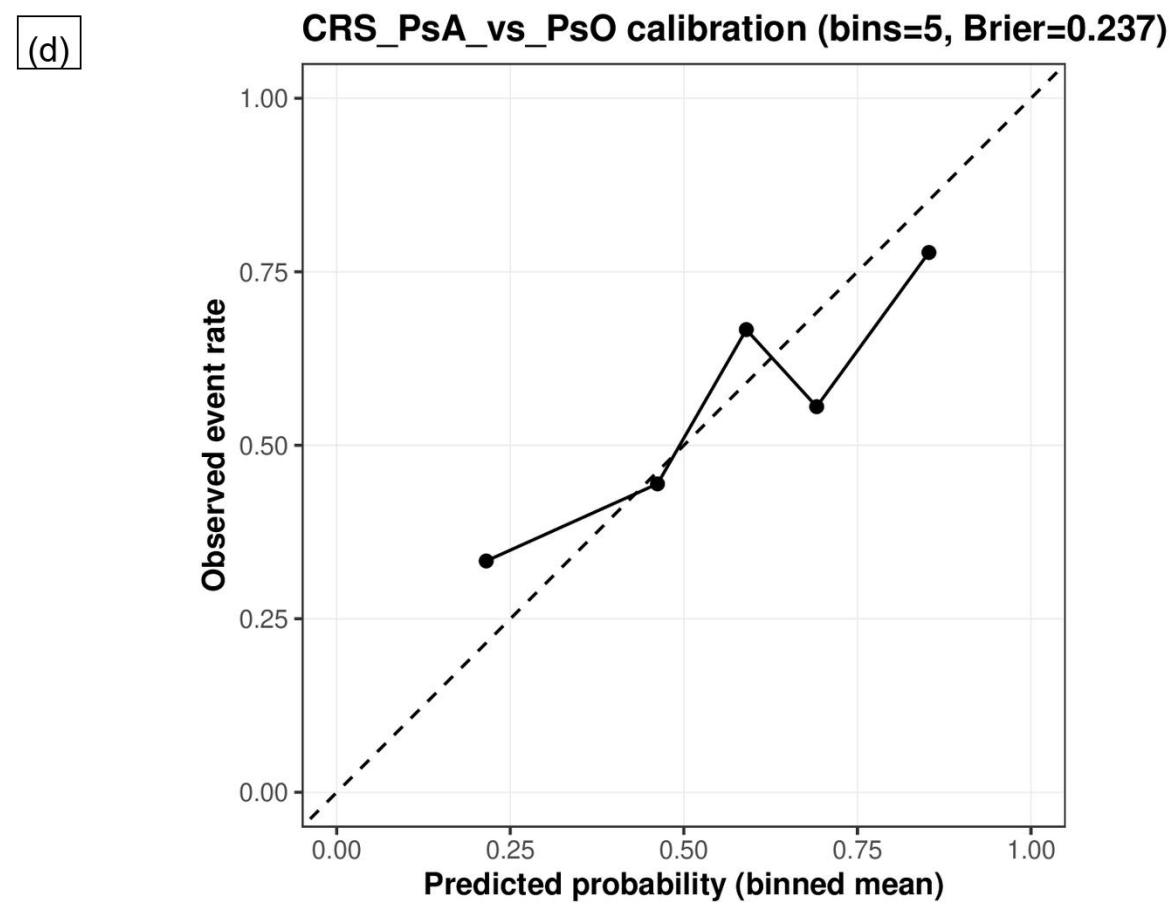

Supplement: Supplementary file 1 [file ijms-27-05121-s001.zip › supplementary_ijms_v8_tex_package/Definitions/Supplementary_Figure_S1_GSE200376_Performance.pdf]

# Untreated-analysis-set CRS\_norm map across all eligible annotated cell types

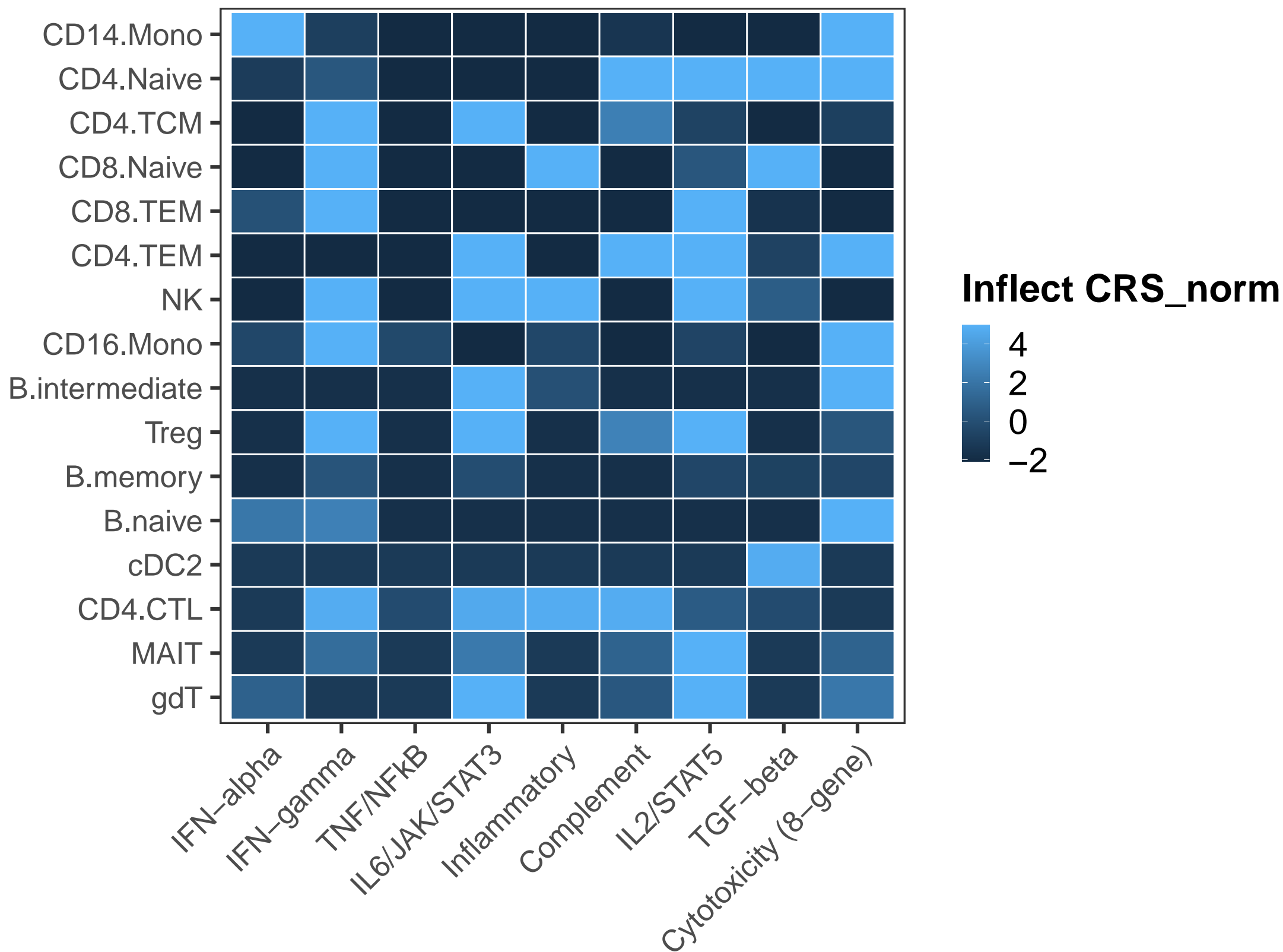

Supplement: Supplementary file 1 [file ijms-27-05121-s001.zip › supplementary_ijms_v8_tex_package/Definitions/Supplementary_Figure_S7_GSE194315_AllCell_Exploratory.pdf]

(a)

**DIR vs CRS**  
(GSE236694 CD4 methylation)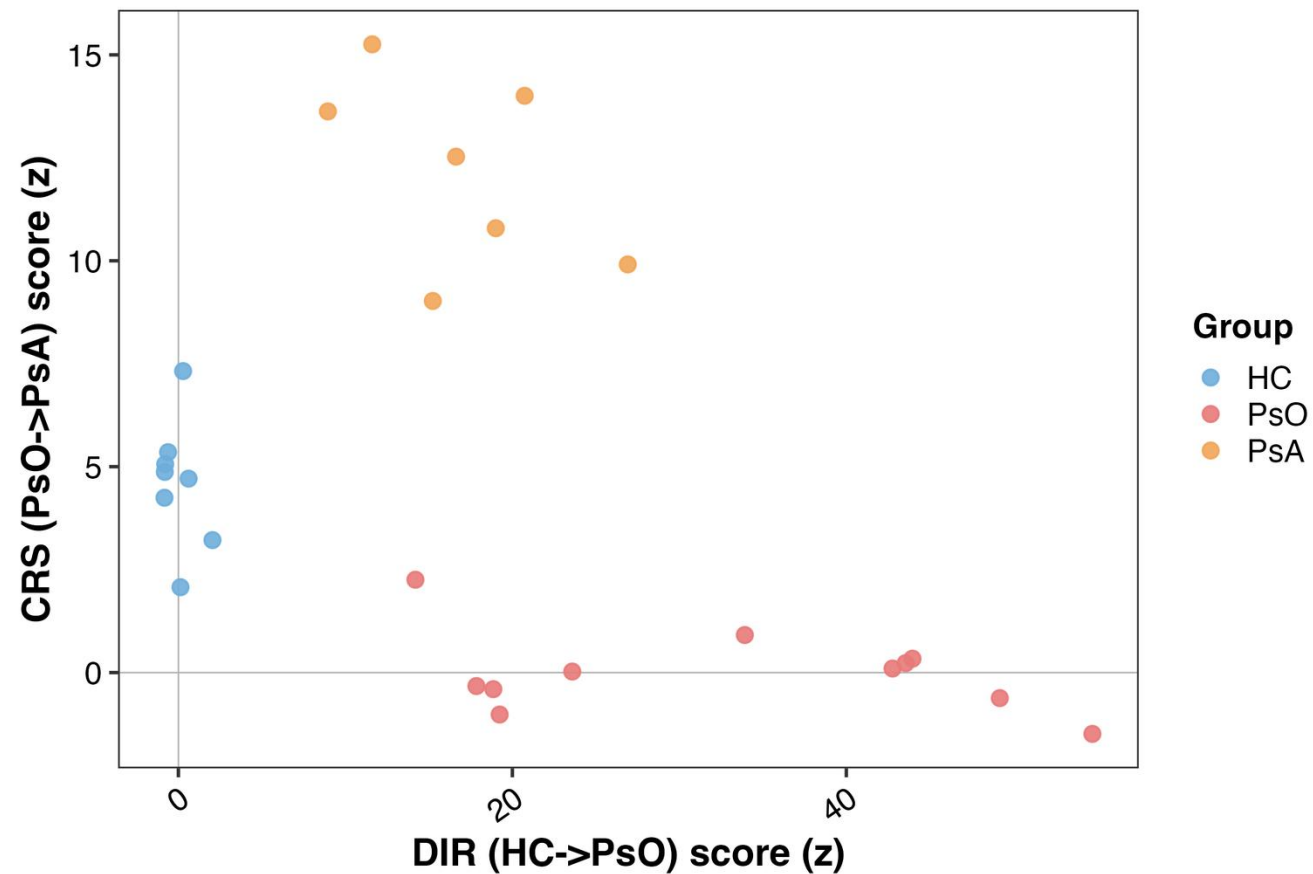

(b)

**DIR/CRS scores**  
(GSE236694 CD4 methylation)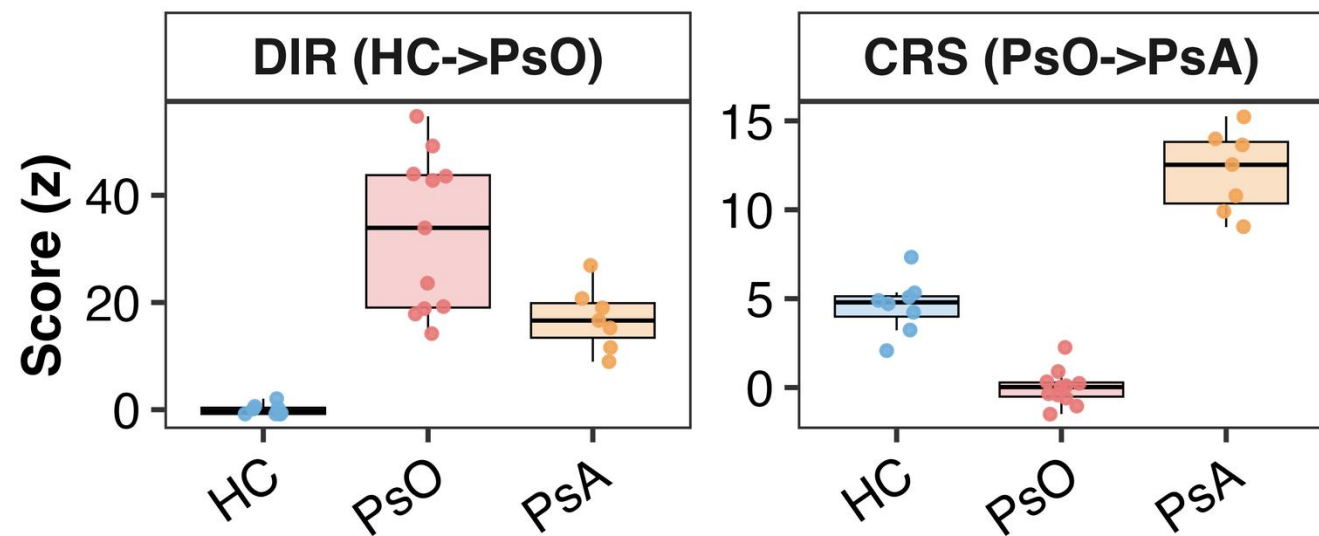

Supplement: Supplementary file 1 [file ijms-27-05121-s001.zip › supplementary_ijms_v8_tex_package/Definitions/Supplementary_Figure_S4_GSE236694_CD4_Methylation.pdf]
